# Efficiency of Primary Health Services in the Greek Public Sector: Evidence from Bootstrapped DEA/FDH Estimators

**DOI:** 10.3390/healthcare12222230

**Published:** 2024-11-08

**Authors:** Angeliki Flokou, Vassilis H. Aletras, Chrysovalantis Miltiadis, Dimitris Charalambos Karaferis, Dimitris A. Niakas

**Affiliations:** 1 School of Social Sciences, Hellenic Open University, 26335 Patra, Greece; valetras@uom.edu.gr (V.H.A.); chrysmilt@gmail.com (C.M.); dimitris.niakas@gmail.com (D.A.N.); 2 Department of Business Administration, University of Macedonia, 54636 Thessaloniki, Greece; 3 Medical School, National and Kapodistrian University of Athens, 11527 Athens, Greece; karafedis@yahoo.gr

**Keywords:** primary healthcare (PHC), efficiency, data envelopment analysis (DEA), free disposal hull (FDH), super-efficiency, bootstrap, Greek public sector

## Abstract

Strengthening primary healthcare (PHC) is vital for enhancing efficiency and improving access, clinical outcomes, and population well-being. The World Health Organization emphasizes the role of effective PHC in reducing healthcare costs and boosting productivity. With growing healthcare demands and limited resources, efficient management is critical. **Background/Objectives:** Building on this point, this study aimed to evaluate the efficiency of PHC units across Greece, focusing on Health Centers (HCs) and Local Health Units (ToMYs). The objective was to assess their efficiency levels and identify factors contributing to observed inefficiencies. This study explores a novel research area by being the first to assess the efficiency of restructured primary healthcare facilities in Greece, utilizing 2019 data—the first year operational data became available for the newly established ToMY facilities following recent healthcare reforms. **Methods:** We applied a comprehensive suite of non-parametric methods, including Data Envelopment Analysis (DEA) under variable, constant, increasing, and decreasing returns to scale (VRS, CRS, IRS/NDRS, DRS/NIRS) assumptions, along with the Free Disposal Hull (FDH) model, all oriented toward output maximization. Efficiency scores were refined using bootstrapping to calculate 95% confidence intervals, and efficient units were ranked via the super-efficiency model. Outliers were identified and removed through the data cloud algorithm. For the first time at this scale, the final sample included the vast majority of PHC facilities in Greece—234 Health Centers and 94 Local Health Units—with inputs categorized into three human resource types: medical, nursing/paramedical, and administrative/other staff. Outputs encompassed scheduled visits, emergency visits, and pharmaceutical prescription visits. This diverse and comprehensive application of DEA methods represents a novel approach to evaluating PHC efficiency in Greece, with potential relevance to broader healthcare contexts. **Results:** The analysis revealed significant inefficiencies and differences in technical efficiency between HCs and ToMYs. HCs could nearly double their outputs (VRS score: 1.92), while ToMYs could increase theirs by 58% (VRS score: 1.58). Scale efficiency scores were closer, with HCs slightly more aligned with their optimal scale (1.17 vs. 1.20 for ToMYs). **Conclusions:** There is significant potential to improve efficiency in PHC, with variations depending on unit characteristics and regional differences. This evaluation provides a foundation for policymakers to identify areas for improvement and enhance the overall performance of healthcare services in Greece.

## 1. Introduction

As stated in the Astana Declaration, primary healthcare (PHC) is the cornerstone of health systems, essential for achieving universal health coverage and meeting the health-related Sustainable Development Goals of the United Nations [1]. According to the World Health Organization [2], PHC aims to ensure the highest possible level of health and well-being for society based on people’s needs and encompasses a wide range of services, from information and prevention to treatment and rehabilitation. Strengthening PHC services is a vital investment to improve health equity, access, clinical outcomes, and population well-being while reducing healthcare and social costs and enhancing population productivity [3].

Sufficient funding and the reinforcement of PHC are crucial objectives for health systems as they play key roles in enhancing healthcare efficiency, service quality, resource utilization, and equitable access for all citizens. Historically, PHC in Greece has been marked by a lack of centralized planning and comprehensive policies, leading to fragmented healthcare services; understaffing of specialized personnel; significant shortages in diagnostic equipment, especially in rural areas; limited community-based prevention and health promotion services; poor coordination with secondary care; and high levels of public dissatisfaction [4,5,6,7].

The absence of mandatory referral (gatekeeping) mechanisms from primary to secondary care and the failure to establish a functional connection between PHC services and hospital care result in systemic inefficiencies, further contributing to public dissatisfaction [7]. In 2010, during the economic crisis, PHC became a target for reforms as part of the economic adjustment program. The reforms aimed to reduce and rationalize costs while improving efficiency and effectiveness [8,9,10,11].

Law 3918/2011 [12] established the National Organization for the Provision of Health Services (EOPYY) by merging primary social security funds (IKA, OGA, OAEE, OPAD). Law 4238/2014 [13] introduced the National Primary Care Network (PEDY), which integrated IKA’s PHC services into the National Health System (ESY), transforming EOPYY into a healthcare purchaser, and, additionally, all public PHC facilities were placed under the jurisdiction of the Regional Health Authorities (RHA/YPE) [8,14,15]. Later, Law 4486/2017 [16] renamed PEDY Health Units as Health Centers and established Local Health Units (ToMYs) as decentralized branches of these Health Centers, aiming to provide holistic, efficient, and patient-centered care tailored to the population’s needs.

Currently, in Greece, primary healthcare (PHC) services in the public sector are provided through structures of the Greek National Health System (ESY), including Health Centers (HCs) and their affiliated Regional Medical Offices, Multipurpose Regional Medical Offices, Specialized Regional Medical Offices, Local Medical Offices, Local Health Units (ToMYs), and outpatient clinics in hospitals [17].

Between 2010 and 2019, there was a 24.5% decrease in medical personnel and a 29.5% reduction in non-medical personnel in Health Centers, contrasted by a 26.6% increase in nursing personnel [17,18]. Ambulatory care expenditures decreased by 41.68%, with public spending down by 21.03% and private spending down by 52.25%, reflecting declining disposable incomes [19,20,21,22]. Despite these reductions, private expenditures still constituted a larger share than public spending.

As healthcare needs grow and resources remain constrained, efficient management becomes crucial. Modern methods for assessing the efficiency of PHC units, such as Data Envelopment Analysis (DEA), offer valuable tools for policymakers. DEA is a non-parametric method widely used to evaluate the efficiency of healthcare units, including hospitals and PHC facilities [23,24,25,26,27,28,29,30,31,32,33].

### 1.1. Related Studies

In Greece, studies utilizing Data Envelopment Analysis (DEA) have primarily focused on evaluating the efficiency of primary health facilities, namely, the NHS’s Health Centers (HCs) and IKA’s healthcare centers. [4,34,35,36,37]. One early study [35] analyzed 133 of the 242 IKA’s healthcare centers, which, at that time, served as the primary public health insurance provider. The analysis employed output data from 1998 (including patient visits and laboratory tests) and staffing data from September 1999. The results were categorized by the population served, with groups defined as up to 10,000, 10,000–25,000, 25,000–50,000, and over 50,000. The findings indicated that units equipped with technological resources for laboratory and radiological examinations demonstrated higher efficiency. Moreover, mid-sized units performed better than larger or smaller units. IKA HCs serving populations between 10,000 and 50,000 individuals were notably efficient, with none in the 25,001–50,000 range scoring below 50% efficiency.

Another study [4] evaluated the efficiency of 194 primary healthcare facilities using data from 2004, which included 103 NHS Health Centers (HCs), representing 67.9% of the total, and 91 IKA healthcare centers, representing 57.8% of the total. The facilities were categorized by the population served into three groups: small (<15,000), medium (15,000–30,000), and large (>30,000). Additionally, they were classified by location as urban/semi-urban or remote/island-based. The results showed that IKA healthcare centers were generally more efficient than NHS Health Centers (84.9% vs. 70.1%). Smaller units outperformed medium and large ones (84.2%, 72.4%, and 74.3%, respectively), and remote/island units were more efficient than urban centers (81.1% vs. 75.7%). In terms of scale efficiency, IKA units again outperformed NHS units (89.7% vs. 85.9%), with large and urban units showing higher efficiency scores (96.3% and 91.9%, respectively) than medium/small and remote units (90.9%–75.9% and 75.3%). These findings suggested a potential influence of facility size, location, and administrative affiliation on primary healthcare efficiency.

A separate study [36] assessed the efficiency of 152 NHS Health Centers using data from 2005. The findings indicated that 61 centers (39%) were technically efficient. Regarding scale efficiency, 96 centers (62.7%) demonstrated increasing returns to scale, while 15 (9.8%) exhibited decreasing returns. The results indicated that larger and urban Health Centers performed more effectively, whereas smaller and rural Health Centers faced scale inefficiencies.

A recent study [34] evaluated the efficiency of 42 Health Centers within the sixth Regional Health Authority, using data from 2010. The average efficiency scores were 0.57 for overall technical efficiency (TE), 0.67 for pure technical efficiency (PTE), and 0.87 for scale efficiency (SE). Only two Health Centers reached full technical efficiency, while nine achieved full pure technical efficiency and five attained full-scale efficiency. The study identified technical inefficiency as the primary source of overall inefficiency, suggesting that these Health Centers could potentially increase output by an average of 33% without additional resources by optimizing current production capacities.

Given these past findings, it is essential to reassess the current efficiency level of Greece’s primary healthcare (PHC) structures to evaluate resource utilization, identify inefficiencies, assess recent reforms, examine regional variations, and inform new policy recommendations.

### 1.2. Application Context

This study, based on 2019 data, offers the only post-crisis, pre-COVID-19 efficiency assessment of Health Centers (HCs) and Local Health Units (ToMYs) in Greece. The year 2019 is significant as it marks the first time operational data became available for the newly established ToMY facilities. Additionally, the number of HCs increased substantially following Law 4486/2017, which reclassified PEDY Health Units as Health Centers and established the ToMY units. The significance of this research is underscored by the ongoing healthcare reforms aimed at implementing a new national health map, with a primary focus on strengthening the PHC sector to reduce pressure on overburdened secondary facilities, conserve resources, and enhance the quality and accessibility of healthcare services.

Identifying the potential for output expansion, specifically the additional capacity for service delivery that Health Centers (HCs) and ToMYs could provide, is a significant goal of this study. This research is the first to quantify the “additional capacity” of the country’s primary healthcare structures using existing resources, employing a comparative evaluation approach.

To our knowledge, this is the only study that (a) includes the largest sample of Health Centers to date, covering over 75% of all centers with full geographic distribution across the country; (b) represents the first analysis of ToMY structures, examining nearly all units; and (c) uses the most recent data. This study’s methodological innovation extends beyond a conventional, naïve application of DEA models, employing a suite of methods to address the inherent limitations of basic DEA applications. This extensive methodological framework provides a detailed presentation of each method’s theoretical underpinnings and implications, equipping managers and policymakers with a deeper understanding of these analytical techniques.

This study addresses potential biases by identifying and excluding outliers and applying the bootstrap method to assess the robustness of results at specific statistical significance levels. Additionally, the super-efficiency method is used to classify efficient units and identify those with unique production profiles that warrant closer examination, while the Free Disposal Hull (FDH) model highlights indisputable benchmark units that outperform others across all dimensions. Furthermore, this study is the first to report results across regions stratified by levels of urbanization, using both Eurostat [38,39] and FAO [40] typologies, and by geographical area as defined by the administrative boundaries of the seven Health Regional Authorities (RHAs/YPE) in the Greek health system.

Key motivations for this approach include (a) assessing efficiency at the national level as well as within distinct geographic areas to determine the potential for additional services within the current resource framework, which could alleviate pressure on secondary care facilities, and (b) identifying individual efficiency scores for targeted interventions at the unit level.

The remainder of this paper is structured as follows: Section 2 details the methods and provides a brief overview of the theoretical background of the techniques employed to measure efficiency, including the software tools used, data selection process, model specification, DEA application, and urbanization and administrative classification levels chosen. Section 3 presents the results of the analysis; Section 4 discusses the findings, limitations, and policy implications; and, finally, Section 5 concludes with a summary and recommendations.

## 2. Materials and Methods

### 2.1. Methods

This research used cross-sectional data from 2019 to assess the relative efficiency of primary healthcare facilities in Greece. This study applied Data Envelopment Analysis (DEA) to benchmark Health Centers (HCs) and Local Health Units (ToMYs) based on resource utilization and service outputs. The non-parametric Data Envelopment Analysis (DEA) method was employed based on the assumptions of the constant returns to scale (CRS), variable returns to scale (VRS), increasing returns to scale (IRS/NDRS), and decreasing returns to scale (DRS/NIRS) models, as well as the Free Disposal Hull (FDH) model, all of them oriented toward output maximization. Efficiency scores were adjusted using a bootstrap technique to derive 95% confidence intervals. A suitable bootstrap method was also utilized to determine the type of global returns to scale for the underlying technology. Comparative evaluation and ranking of the efficient units were performed using the super-efficiency model. Additionally, the results were presented as aggregated scores (weighted averages) across various geographical areas and the seven Regional Health Authorities (RHAs/YPE) in the country and statistically analyzed to identify any significant differences.

#### 2.1.1. Data Envelopment Analysis (DEA)

Data Envelopment Analysis (DEA) is a non-parametric method introduced by Charnes et al. in 1978 [41]. It utilizes linear programming to benchmark the efficiency of a group of similar production units, known as decision-making units (DMUs). These DMUs use a common set of inputs to produce a common set of outputs. The evaluation is relative, meaning that units showing no potential for improved efficiency are assigned a score of 1, indicating 100% efficiency. For all other units, a score below or above 100% is assigned. This score reflects, for each unit, the percentage by which inputs must be reduced (while maintaining current output levels) or the percentage by which outputs must be increased (while maintaining current input levels) to achieve 100% efficiency. In the former case, the efficiency is input-oriented, and, in the latter, it is output-oriented.

In geometric terms, each production unit can be represented as a point within the input–output space. Units with the same output levels can be compared based on their distance from the origin if they are positioned along the same radial direction in the input space. The unit closest to the origin is the most efficient, receiving an efficiency score of 1 (or 100%), while units farther from the origin are less efficient, with scores below 100%. This efficiency score is calculated as the ratio of the distance of the most efficient unit to the origin compared to that of the less efficient unit. Similarly, for units with the same input levels along the same radial direction in the output space, the unit farthest from the origin is deemed the most efficient, also receiving a score of 1. In this case, units closer to the origin have efficiency scores exceeding 100%. This score is determined by the ratio of the efficient unit’s distance from the origin to that of each less efficient unit. The assessment of efficiency mentioned above relies on what is known as the Farrell measure of efficiency [42].

However, this method can only be applied when there are at least two units along the same radial direction in the input or output space, sharing identical input or output levels. Only then is it possible to compare their distances and calculate efficiency scores. However, in practice, given that samples under evaluation consist of only a limited, discrete number of production units, it is highly improbable that units will align perfectly along the same radial direction in the input or output space, considering the infinite number of possible directions. To enable meaningful comparisons, it becomes necessary to determine the most efficient point along each radial direction.

This can be achieved by applying certain assumptions that allow transitioning from the discrete set of production possibilities in the sample to a complete (dense) set. From this full set, the most efficient operating point for each radial direction is identified. Geometrically, this corresponds to defining the feasible production possibilities region and, consequently, its outer boundary, known as the efficiency or best-practice frontier, which separates feasible from infeasible production possibilities. The points on this frontier represent the most efficient units. The intersection of this surface with the radial directions of the units being evaluated identifies the benchmarks, against which the efficiency scores of other units can be measured. The assumptions that are typically made include the assumption of the free disposability of inputs and outputs, the convexity of the production possibility set, and the type, if any, of prevailing returns to scale (non-increasing, non-decreasing, or constant). The construction of the production possibility set ***P*** under the aforementioned assumptions is derived mathematically as follows [43,44]:

Consider a set of *n* decision-making units (DMUs), where each unit ***p*** utilizes varying amounts of different *m* inputs

$$x_{1p},x_{2p},\cdots ,x_{mp}$$

and produces varying amounts of the total of s outputs

$$y_{1p},\;y_{2p},\;\cdots ,y_{sp}$$

where inputs and outputs are considered non-negative, without all being zero (i.e., at least one input and at least one output are strictly positive numbers) [45]:

$$x_{ip\;}\geq 0,\;y_{jp\;}\geq 0\;\forall \;i,j,p$$

$$\sum_{i=1}^{m}x_{ip}>0,\;\sum_{j=1}^{s}y_{jp}>0$$

Each such decision-making unit p is called an activity and will henceforth be generally denoted by the pair $\left(x,y\right)$ or the pair $\left(x_{r},y_{r}\right)$ (when there is a need to refer to the specific index r that indicates a particular DMU).

Starting from the set of the n total units mentioned above, for which the corresponding n productive activities are (empirically) known

(1) $$\left(x_{r},y_{r}\right),\;\left(r=1,2,\ldots ,n\right)$$

a more comprehensive set of feasible production possibilities is constructed by incorporating all additional production possibilities derived from one or more of the following assumptions:

▪ Assumption of Free Disposability of Inputs and Outputs: If an activity $\left(x,y\right)$ belongs to ***P***, then any activity $\left(x^{\prime },y^{\prime }\right)$ with $x^{\prime }\geq x$ and $y^{\prime }\leq y$ will also belong to ***P***.
▪ Assumption of Convexity of Set ***P***: Any weighted average of activities in ***P*** also belongs to ***P***
  (2) $$\left(x,y\right)\in P,\;\left(x^{\prime },y^{\prime }\right)\in P\;\Rightarrow \;\lambda \left(x,y\right)+\left(1-\lambda \right)\left(x^{\prime },y^{\prime }\right)\in P,\;\lambda \in \left[0,1\right]$$
▪ Assumption of the Type of Returns to Scale: If an activity $\left(x,y\right)$ belongs to ***P*** then the activity $\left(\lambda x,\lambda y\right)=\lambda \left(x,y\right)$ also belongs to ***P***, where
  i. $\lambda \in \left(0,1\right]$ under the assumption of non-increasing returns to scale (NIRS)
  ii. $\lambda \in \left[1,+\infty \right)$ under the assumption of non-decreasing returns to scale (NDRS)
  iii. $\lambda \in \left(0,+\infty \right)$ under the assumption of constant returns to scale (CRS)

Based on these assumptions, the production possibility set ***P*** is given by the following construct:

(3) $$P^{PPS}=\left\{\left(x,y\right){\in \;R}_{+}^{m}\times R_{+}^{s}|\;\exists \;\lambda_{r}\in \Lambda (PPS):\;x\geq \sum_{r=1}^{n}\lambda_{r}x_{r},\;y\leq \sum_{r=1}^{n}\lambda_{r}y_{r}\right\}$$

where *PPS* values are given by

$$PPS\in \left\{CRS,\;NIRS,NDRS,VRS,\;FDH\right\}$$

and the sets Λ(*PPS*) are defined according to the specifications provided in Table 1 below:

The input-oriented (-IO) efficiency of unit ***p*** is calculated by solving the next linear programming problem

(4) $${TE}_{p}^{PPS-IO}=min\left\{\theta_{p}:\theta_{p}x_{p}\geq \sum_{r=1}^{n}\lambda_{r\left(p\right)}x_{r},\;y_{p}\;\leq \;\sum_{r=1}^{n}\lambda_{r\left(p\right)}y_{r},\;\lambda_{r\left(p\right)}\in \Lambda \left(PPS\right)\right\}$$

whereas the output-oriented efficiency (-OO) is derived respectively by the following problem:

(5) $${TE}_{p}^{PPS-OO}=max\left\{\Phi_{p}:x_{p}\geq \sum_{r=1}^{n}\lambda_{r\left(p\right)}x_{r},\;y_{p}\Phi_{p}\;\leq \;\sum_{r=1}^{n}\lambda_{r\left(p\right)}y_{r},\;\lambda_{r\left(p\right)}\in \Lambda \left(PPS\right)\right\}$$

The assumptions listed in the first three rows of Table 1 correspond to the three DEA models, each defined by their assumption regarding returns to scale: DEA-CRS (constant returns to scale), DEA-NIRS (non-increasing returns to scale), and DEA-NDRS (non-decreasing returns to scale). The model in the fourth row is known as DEA-VRS (variable returns to scale), while the final row represents the FDH (Free Disposal Hull) model. For completeness of terminology, CRS and VRS are also referred to as CCR and BCC [46], with their scores representing overall efficiency and pure technical efficiency, respectively.

#### 2.1.2. Free Disposal Hull (FDH)

The key distinction between FDH and DEA models lies in their assumptions about the production possibility set. In the FDH model [47], the sole assumption is the free disposal of inputs and outputs, meaning that the production possibility set is determined strictly by the actual production activities of the units being evaluated. Consequently, FDH benchmarking is based on comparison with a specific, existing unit rather than a theoretical production activity created from a linear combination of other units. In contrast, DEA benchmarks against a composite unit formed by aggregating portions of multiple existing units.

This characteristic is reflected in the set of possible values, denoted by Λ(FDH). Consequently, the only permissible linear combinations used for benchmarking in the FDH model are those involving a single existing unit with a coefficient of unity. This is because only in this manner does the condition $\left\{\lambda_{r}\in N_{+}|\sum_{r=1}^{n}\lambda_{r}=1\right\}$ hold.

From a managerial perspective, it is important to note that the identification of a set of dominant production units through the presentation of actual implemented production activities that are demonstrably more efficient lends credibility to the inefficiency scores, which often lack such substantiation when reference is made to an abstract frontier [48].

It should also be noted that, specifically for FDH, the calculation of efficiency can be performed analytically, making it unnecessary to solve the linear programming problem. Specifically, the FDH efficiency score of unit *p*, with an input orientation (-IO) or output orientation (-OO), can be calculated by solving the following simple minimax problems [44]:

(6) $${TE}_{p}^{FDH-IO}=\underset{r:\;y_{r}\geq y_{p}}{\min}\;\underset{i=1,2,\ldots ,m}{\max}\frac{x_{ir}}{x_{ip}}$$

(7) $${TE}_{p}^{FDH-OO}=\underset{r:\;x_{r}\leq x_{p}}{\max}\;\underset{j=1,2,\ldots ,s}{\min}\frac{y_{jr}}{y_{jp}}$$

#### 2.1.3. Peer Units

By solving the above linear programming problems for each unit ***p***, the (input or output oriented) efficiency score, ($\theta_{p}$) or ($\Phi_{p}$), is calculated along with the weighting factors $\lambda_{r\left(p\right)}$, which form a linear combination of fully efficient units [44].

(8) $$\left(\sum_{r=1}^{n}\lambda_{r\left(p\right)}x_{r},\;\sum_{r=1}^{n}\lambda_{r\left(p\right)}y_{r}\right)$$

This linearly combined activity corresponds to the most efficient point against which, if present, the evaluated unit p would ultimately be compared. In other words, this is the point on the efficient frontier where the production point under evaluation is radially projected. In this context, the units of the sample that have been assigned positive weighting factors are referred to as the “peer units” of unit ***p***:

(9) $$Peer\;Units\;of\;p=\left\{\left(x_{r},y_{r}\right),\;r\in \;\left\{1,2,\ldots ,n\right\}\;|\;\lambda_{r\left(p\right)}>0\right\}$$

#### 2.1.4. Super Efficiency

A limitation of the DEA evaluation method is that all units forming the efficiency frontier receive a score of 1, making it difficult to rank efficient units. To differentiate between them, the super-efficiency model, introduced by Andersen and Petersen [49], calculates how much a unit can “underperform” while still remaining efficient. This margin is based on either the maximum possible increase in inputs or decrease in outputs until the unit’s score reaches 1. Larger margins indicate greater relative efficiency.

In the super-efficiency model, each unit is excluded from the efficiency frontier calculation. If efficient, the unit’s removal shifts its position, resulting in a score either greater or less than 1. This score allows for the ranking of efficient units based on their distance from the frontier. In output-oriented models, a score below 1 means that the unit can produce more output than other efficient units, with larger deviations indicating higher super-efficiency [49,50,51].

Therefore, the super-efficiency DEA model enhances traditional DEA by ranking efficient units, detecting outliers, identifying untapped potential, and providing more accurate benchmarks for improvement. It offers decision-makers detailed insights into both the robustness and the vulnerabilities of their units, helping them optimize resource allocation, improve operational efficiency, and set more effective performance targets for both efficient and inefficient units.

To compute super-efficiency scores, the two linear programming problems, as well as the sets Λ(CRS), Λ(NIRS), Λ(NDRS), and Λ(VRS), are reformulated, with the sole modification being the exclusion of the unit being evaluated, denoted as *p*. Practically, this means that in all summations where the index *r* ranges from r=1 to r=n, the value corresponding to the evaluated unit *p* is excluded (r≠p).

(10) $$\sum_{r=1}^{n}\left(\ldots \right)\to \sum_{\begin{array}{c} r=1\\ r\neq p \end{array}}^{n}\left(\ldots \right)$$

#### 2.1.5. Weighted Average Efficiency Score

When assessing the efficiency of production units, it is often valuable to consider not only the individual efficiency scores but also the overall efficiency of all units, represented by a comprehensive score for an average representative unit. Using a simple arithmetic mean of the individual efficiency scores can be misleading [52] and, therefore, a weighted mean is recommended. For an output-oriented model with a sample of *n* units using *s* outputs, the weighted mean is calculated as follows:

(11) $${\bar{TE}}_{avg}^{PPS-OO}=\sum_{p=1}^{n}w_{p}{TE}_{p}^{PPS-OO}$$

where the weighting $w_{p}$ of each unit *p* is calculated as

(12) $$w_{p}=\frac{1}{s}\left(\frac{y_{1p}}{\sum_{r=1}^{n}\left\{y_{1r}\right\}}+\frac{y_{2p}}{\sum_{r=1}^{n}\left\{y_{2r}\right\}}+\ldots +\frac{y_{sp}}{\sum_{r=1}^{n}\left\{y_{sr}\right\}}\right)=\frac{1}{s}\sum_{j=1}^{s}\frac{y_{jp}}{\sum_{r=1}^{n}\left\{y_{jr}\right\}}\;p=1,2,\ldots ,n$$

#### 2.1.6. Outliers

Outliers are observations that deviate substantially from the majority of observations in a sample dataset, either being much higher or lower than typical values, often falling outside the “expected range” of the data. These anomalies may result from natural variability, measurement errors, or rare occurrences and can significantly influence the accuracy of models, particularly in the context of Data Envelopment Analysis (DEA), where they can distort efficiency estimates and skew results. Outliers in a DEA dataset can significantly distort the construction of the corresponding best-practice frontier, which, in turn, can heavily influence the efficiency scores of certain units. As such, it is crucial to identify and remove these outliers. Although there are several methods for detecting outliers in non-parametric frontier models, no single method can identify all outliers under any circumstances. For this reason, it is often recommended to use multiple methods when analyzing data. In this study, the data cloud method, as outlined by Wilson [53], was chosen as the primary approach for detecting outliers. This method assesses the geometric volume D formed by the DMUs and analyzes how sensitive this volume is to changes when subsets of DMUs—such as individual units, pairs, or triplets—are excluded from the dataset. A group of *r* DMUs are considered potentially outlying if their removal results in a significant change in the volume.

#### 2.1.7. Returns to Scale Assumption

In this study, the method proposed by Simar and Wilson [54] was utilized to identify the appropriate returns to scale type. This approach, which employs bootstrapping, consists of two sequential tests. The first test (test 1) evaluates the null hypothesis that the production function has globally constant returns to scale (CRS) against the alternative hypothesis that the technology exhibits globally variable returns to scale (VRS):

Test 1: [H_0_: technology is globally CRS] versus [H_1_: technology is VRS].

If H_0_ is rejected, the second test is conducted. Test 2 assesses whether the technology displays globally non-increasing returns to scale (NIRS) compared to the alternative of globally VRS:

Test 2: [H’_0_: technology is globally NIRS] versus [H’_1_: technology is VRS].

If both H_0_ and H’_0_ are rejected, this indicates that the underlying technology demonstrates globally variable returns to scale (VRS). Among the various test statistics available for these tests, the following ratios of sums (across all units) were chosen for application:

(13) $${ts}_{1}=\frac{\sum_{i=1}^{n}{TE}_{i}^{CRS}}{\sum_{i=1}^{n}{TE}_{i}^{VRS}},\;{ts}_{2}=\frac{\sum_{i=1}^{n}{TE}_{i}^{NIRS}}{\sum_{i=1}^{n}{TE}_{i}^{VRS}}$$

These two statistics can be interpreted as representing the ratios of the average distances from CRS to VRS frontiers (for test 1) and from NIRS to VRS frontiers (for test 2).

#### 2.1.8. Scale Efficiency: Most Productive Scale Size (MPSS)

The Most Productive Scale Size (MPSS) is defined [55] as a production possibility $\left(x_{0},y_{0}\right)\in P$ whose average productivity of its input–output mix is maximized. At the MPSS, the average output is at its peak, and any deviation from this point of operation results in reduced average output. Formally, $\left(x_{0},y_{0}\right)\in P$ is an MPSS for its input and output mix if, and only if, for all $\left({\alpha x}_{0},{\beta y}_{0}\right)\in P\Rightarrow \;\alpha \geq \beta$

The loss from not operating at the MPSS is quantified by scale efficiency (SE), which is calculated as

(14) $$SE=\frac{{TE}^{CRS}}{{TE}^{VRS}}$$

The relationship between MPSS and scale efficiency is captured by the following proposition: A production is an MPSS for its input and output mix if, and only if, ${TE}^{VRS}\;$= 1 and SE=1. Therefore, if $\left(x_{0},y_{0}\right)$ is an MPSS, then constant returns to scale prevail at $\left(x_{0},y_{0}\right)$.

The Most Productive Scale Size (MPSS) is the point where constant returns to scale prevail, and it reflects how close a unit is to achieving its optimal scale of operation. Units with increasing returns to scale (IRS) fall before the MPSS, while those with decreasing returns to scale (DRS) come after it on the efficiency frontier. It is important to note, however, that the MPSS is specific to the particular input and output mix being evaluated.

#### 2.1.9. Bootstrap

A notable criticism of DEA is its underlying assumption that the entire distance of a DMU from the efficiency frontier is attributed solely to inefficiency. In practice, this distance also captures noise, as input and output data are typically prone to errors. Since DEA uses non-statistical linear programming methods, it cannot evaluate the accuracy of the efficiency estimates. Additionally, the estimated production technology is only a partial representation of the true, unknown technology, meaning that DEA efficiency scores tend to be higher than the actual values. This inherent upward bias in DEA scores is a structural issue. To mitigate this problem, Simar and Wilson [56,57] proposed a bootstrap-based methodology that helps assess the statistical properties of DEA estimators. In this study, their bootstrapping method was applied to compute bias-corrected efficiency scores and provide estimates of their confidence intervals.

**Figure 1:**
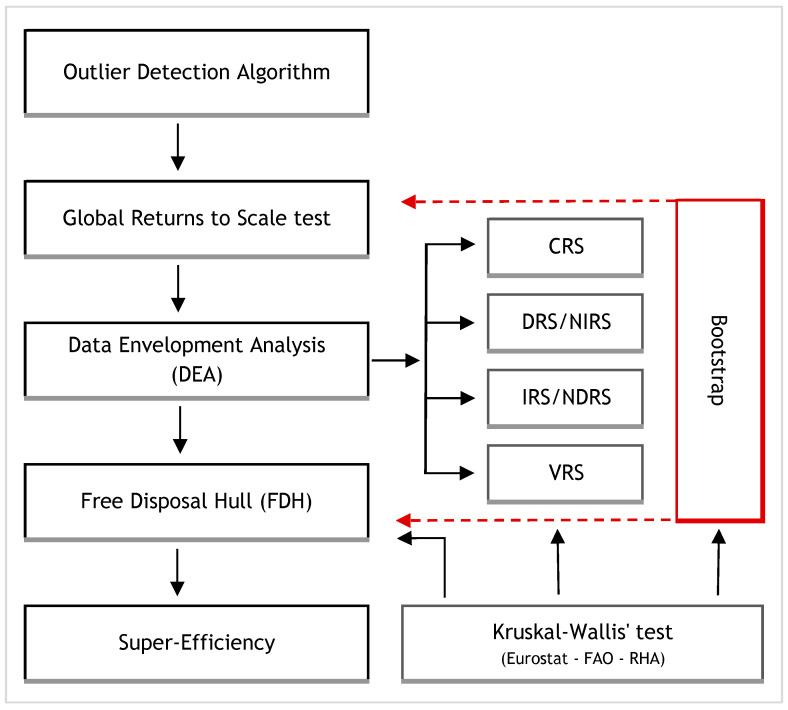
Workflow of methods used for efficiency analysis.

Figure 1 presents the methodological workflow for the efficiency analysis, including outlier detection, returns-to-scale testing, DEA with various scale assumptions, super-efficiency, FDH, statistical validation via bootstrap, and statistical inference using Kruskal–Wallis tests.

### 2.2. Software Tools

For this study, existing software applications were used to solve specific models (EMS, DEA SOLVER, R packages—“Benchmarking” and “FEAR”) and SPSS 29.0 (IBM Corp., Armonk, NY, USA) for statistical tests. Additionally, custom Excel routines were developed where needed.

### 2.3. Data and Model Specification

#### 2.3.1. Sample

The study sample comprised Health Centers and ToMYs from 2019. Primary data were sourced from the Ministry of Health (following a formal request) for all facilities, totaling 307 Health Centers and 117 ToMYs nationwide. Facilities with incomplete input/output data (66 Health Centers and 17 ToMYs) were excluded, resulting in a sample of 241 Health Centers and 100 ToMYs, representing 78.5% and 85.5% of the total units, respectively.

**Figure 2:**
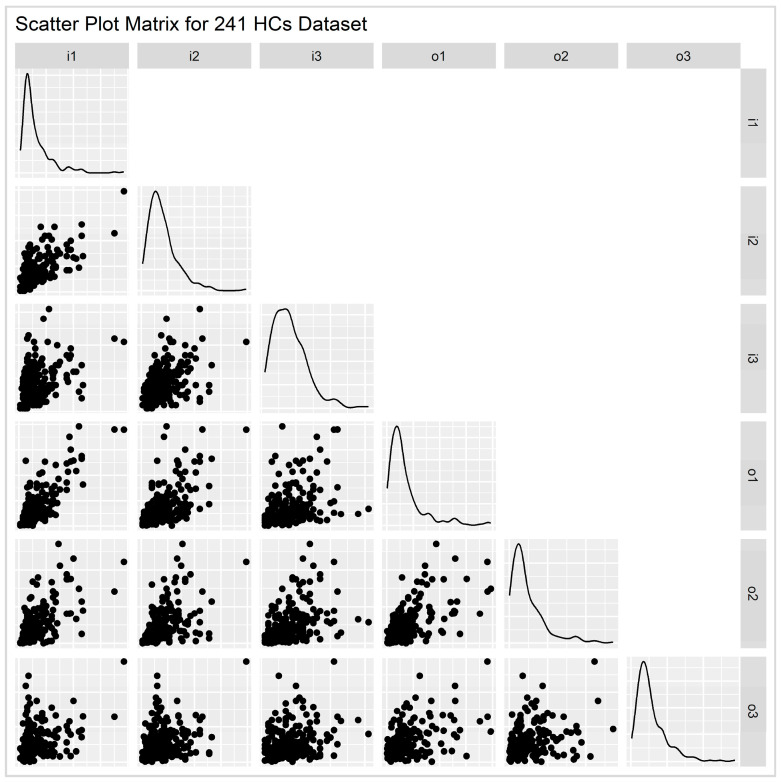
Scatter plot matrix of the three inputs and three outputs for the 241 HCs dataset. Pairwise relationships (in the off-diagonal plots) and individual variable distributions (on the main diagonal) are shown. Inputs: (i1) number of medical staff, (i2) number of nursing and paramedical staff, (i3) number of administrative and other staff. Outputs: (o1) number of scheduled patient visits, (o2) number of emergency patient visits, (o3) number of patient visits for obtaining pharmaceutical prescriptions.

The data were then examined through distribution analysis and pairwise correlations, as illustrated in the scatter plot matrix in Figure 2. A preliminary review of these plots revealed the presence of outlier units, which, as discussed extensively in a later section, were excluded from the analysis to ensure that the remaining data were representative and robust for subsequent analysis. Using an appropriate outlier detection algorithm, 7 Health Centers and 6 ToMYs were identified and removed, resulting in a final sample of 234 Health Centers and 94 ToMYs, representing 76.2% and 80.3% of the original totals, respectively. These sample sizes were considered more than satisfactory, offering both comprehensive and current data coverage with extensive geographic representation across all regions of the country.

**Figure 3:**
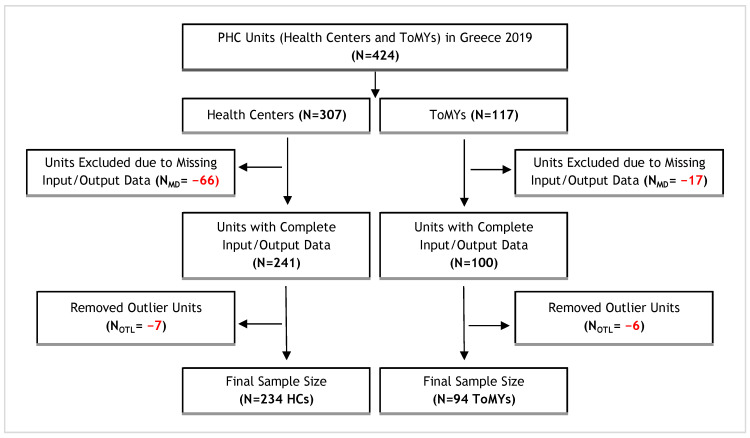
Data selection process for primary healthcare (PHC) units (Health Centers and ToMYs) in Greece, 2019.

The flowchart in Figure 3 outlines the steps to derive the final sample sizes for Health Centers (HCs) and Local Health Units (ToMYs), beginning with the initial dataset of 424 units. Units were excluded due to missing input/output data, and additional outliers were removed, resulting in a final sample of 234 HCs and 94 ToMYs.

#### 2.3.2. Inputs–Outputs

As evidenced in the pertinent literature, the appropriate selection of inputs and outputs is of paramount importance for ensuring the validity and reliability of results when utilizing Data Envelopment Analysis for benchmarking purposes.

Measuring efficiency in primary healthcare (PHC) is particularly challenging due to its holistic role, providing long-term, continuous support to individuals and families [32]. Unlike hospitals, where admissions and discharges create defined boundaries, PHC operates as an open, community-based system with fluid and often indefinite service parameters, complicating economic modeling [58]. Due to limited data on long-term health outcomes, researchers frequently adopt “activity-oriented” models, using metrics such as counts of visits or consultations, vaccinations, prescriptions, minor surgeries undertaken, referrals, and so on as proxies for output [33,59]. In Greece, assessing PHC efficiency is further complicated by variability in service delivery and the absence of a standardized framework. Greek PHC services vary widely, with little consensus on standardized definitions for resources, procedures, or outputs [34]. Different stakeholders often hold conflicting views on what constitutes effective care, while ambiguity in targets and resource definitions further complicates efficiency assessments. Consequently, input and output selection for benchmarking Greek PHC must be meticulously adapted to capture this variability and complexity.

In this study, the inputs selected for benchmarking Greek PHC facilities included (i1) medical staff, (i2) nursing and paramedical staff, and (i3) administrative and other support staff, each representing a distinct component of healthcare resources. The outputs measured were (o1) scheduled patient visits, (o2) emergency patient visits, and (o3) pharmaceutical prescription visits, capturing key activities and service demands. This selection aimed to provide a robust and contextually relevant measure of efficiency, mindful of the unique complexities and operational variability inherent in Greek primary healthcare.

The selected variables in this study were deemed to sufficiently represent the production process of PHC services and were consistent with those employed in similar studies [4,33,34,35,36,59,60,61,62], as shown in Table 2.

#### 2.3.3. Discriminatory Power

With respect to discriminatory power, this study fully satisfied the empirical rule for sample adequacy in DEA. According to this rule of thumb [63], the number of decision-making units (DMUs) should exceed either three times the sum of inputs and outputs or twice their product. With three inputs and three outputs, the minimum threshold qs 18 DMUs (3 × (3 + 3) = 18, 2 × (3 × 3) = 18). The samples analyzed—241 Health Centers (HCs) and 94 Local Health Units (ToMYs)—exceeded this requirement by factors of 13 and 5.4, respectively, providing the DEA model with sufficient discriminatory power to avoid result distortion and minimize the likelihood of misclassifying inefficient units as efficient.

To rank fully efficient units, the super-efficiency model was applied [49] as an indirect method of enhancing the discriminatory capability of standard DEA. Although Principal Component Analysis (PCA) could potentially reduce the number of variables—and thereby decrease the number of units deemed efficient, enhancing discrimination [64]—this approach was not selected for several reasons. First, it was essential to retain all relevant variables to capture the unique contributions of each. The three selected input variables represented distinct components of human resources, each included in disaggregated form due to their complementary yet fundamentally different roles. Second, even after reducing the number of fully efficient units, a subset of efficient units would still require relative ranking. Third, and most importantly, PCA+DEA has inherent limitations: PCA often produces both positive and negative component values, which are incompatible with DEA’s requirement for non-negative data. A workaround—adding the absolute value of the minimum component plus one to each value [65]—would require the DEA models to be translation-invariant. However, this condition was not met by the CRS model, and in the output-oriented VRS model, translation-invariance applied only to the input side [66]. Consequently, in this study, PCA could only be applied to reduce the input dimensions by one, as the cumulative variance of the first two principal components accounted for 86.9% of the total. This reduction would have altered the input space while leaving the output space unchanged, complicating meaningful interpretation and, most importantly, limiting comparability with standard (non-PCA-based) DEA models from other studies, whether past or future.

Moreover, our results, presented in the next section, reveal substantial inefficiencies across DMUs, with mean efficiency scores indicating room for improvement. Given these findings, further refinement to increase discriminative power is unnecessary at this stage. Such adjustments may become relevant in future analyses, once the primary inefficiencies have been addressed.

#### 2.3.4. Orientation

In terms of selecting between input minimization (reflecting a financial perspective) or output maximization (reflecting a social perspective), an output-oriented approach was chosen to identify potential areas for improvement, given the well-documented challenges of resource scarcity and the increasing demand for primary health services. In this context, it is crucial to evaluate current service levels relative to the available inputs. Recent years have seen a focus on strengthening primary care services for prevention and health promotion at the community level, with the goal of progressively developing an integrated healthcare model. Thus, assessing the capacity to expand service provision within the existing network of Health Centers (HCs) and Local Health Units (ToMYs) is essential for any future system redesign. Moreover, it is widely acknowledged that these healthcare units inherently aim to maximize population coverage, with a significant portion of their activities focusing on health promotion, disease prevention, and control. The objective, therefore, is to serve a larger proportion of the population with the available resources, rather than maintaining the same output levels with fewer resources [34,59,61,62,67,68,69,70,71].

### 2.4. Classification by Urbanization Levels

Most of the results for the Health Centers (HCs) will be presented based on urbanization levels, classified according to both Eurostat [38,39] and FAO [40] typologies, as well as their distribution across Regional Health Authorities (RHAs), whereas the results for the Local Health Units (ToMYs) will be shown solely according to their division among the RHAs.

Eurostat and FAO both classify urbanization levels into three categories, but with different criteria. Eurostat categorizes regions with over 1500 inhabitants per square kilometer as urban (‘1’), those with 300–1500 inhabitants per square kilometer as intermediate (‘2’), and those with fewer than 300 inhabitants per square kilometer as rural (‘3’). FAO classifies urban areas as settlements with over 5000 people, peri-urban areas as those with 1000 to 5000 people, and rural areas as having fewer than 1000 people, typically focused on agriculture. Both classifications aim to analyze population distribution and inform planning and development strategies.

## 3. Results

### 3.1. Input–Output Summary Statistics

**Figure 4:**
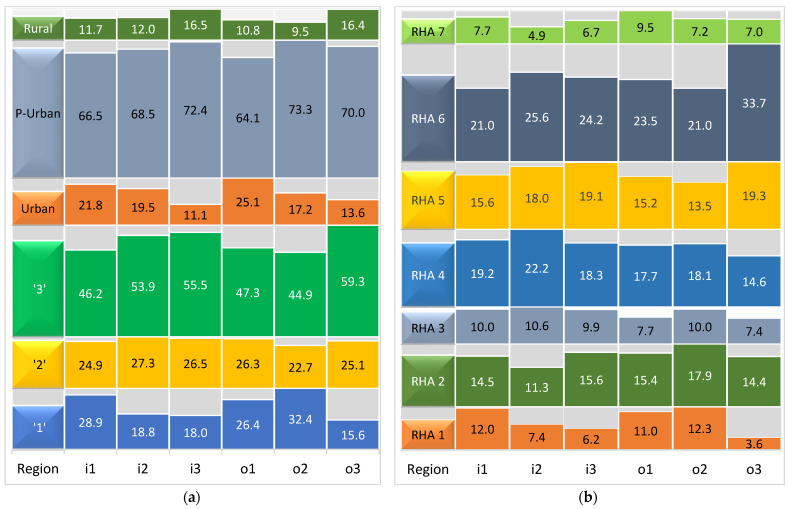
Percentage distribution of Health Center (HC) inputs and outputs across regions categorized by (**a**) Eurostat’s typology (‘1’, ‘2’, ‘3’) and FAO’s classification (rural, peri-urban, urban) and (**b**) Regional Health Authorities (RHA 1-7).

Descriptive statistics of the input and output variables for Health Centers (HCs) and Local Health Centers (ToMYs) that were included in the final sample are presented in Table 3. For HCs, which included 234 units, the mean values indicated that the average HC employs 11.4 medical staff, 15.7 nursing/paramedical staff, and 8.2 administrative personnel. In terms of outputs, the average HC manages 15,992.4 scheduled visits, 9255.6 emergency visits, and 10,409.4 prescription-related visits. The high standard deviations across inputs and outputs suggested significant variation among HCs, with larger centers vastly outstripping smaller ones. For instance, while the median number of scheduled visits was 11,917, the maximum reached a notable 75,381 visits, indicating the presence of some very large centers. This was further evidenced by the range values, with scheduled visits varying by as much as 75,040 across different HCs. The percentage distribution of inputs and outputs across regions, categorized by Eurostat’s classification (‘1’, ‘2’, ‘3’) and FAO’s classification (rural, p[eri]-urban, urban), as well as by Regional Health Authorities (RHA 1-7), are depicted in Figure 4.

In contrast, ToMYs, which consist of 94 units, operate on a smaller scale. The average ToMY employs 2.6 medical staff, 3.1 nursing/paramedical staff, and 2.5 administrative staff. The output averages for scheduled visits (4944.7), emergency visits (1025.9), and prescription-related visits (2860.5) are significantly lower than those for HCs. The lower standard deviations and range values for ToMYs reflected more consistency and less variability among these units, suggesting that they are relatively homogeneous in terms of staffing and patient visits. The maximum number of scheduled visits for ToMYs was 14,161, significantly lower than for HCs, implying a smaller operational scope.

Key observations include the stark difference in scale between the two types of healthcare entities, with HCs serving many more patients and employing larger staff teams. HCs also show much greater variability, as indicated by the larger standard deviations and the presence of outliers, especially for scheduled patient visits. ToMYs, though smaller, still contribute substantially in terms of patient care, particularly in terms of prescription visits. The sum values reveal that across all units, HCs manage a total of about 3.7 million scheduled visits, compared to 464,801 for ToMYs, further illustrating the scale disparity. In summary, the statistical data in the table underscore the distinct operational profiles of the larger, more diverse Health Centers (HCs) compared to the smaller, more homogenous Local Health Units (ToMYs). These differences justified treating them as two separate groups in our analysis, as their underlying technologies appeared to differ significantly.

### 3.2. Global Returns to Scale

Regarding the type of global returns to scale, the application of Simar and Wilson’s two-part test (Table 4) resulted in the rejection of both the H_0_ (ts = 1.1526, *p*-value < 0.001) and H’_0_ (ts = 1.0256, *p*-value = 0.005) null hypotheses at the 1% significance level, following 2000 bootstrap replications. Consequently, variable returns to scale (VRS) was considered the appropriate returns to scale type for the underlying technology of the Health Centers under evaluation. Nonetheless, some results under the constant, non-increasing, and non-decreasing returns to scale (CRS, NIRS, NDRS) assumptions are also provided for comparison and completeness.

### 3.3. VRS Results

Table 5 presents DEA VRS results for HCs across various regions, highlighting their pure technical efficiency. The scores indicate how much the output of each unit could be expanded using the same level of inputs, with values closer to 1 representing higher efficiency. The table also includes mean values for uncorrected and bootstrapped (bias-corrected) efficiency scores, along with weighted means, offering insight into the efficiency across different regions and health authority areas.

**Figure 5:**
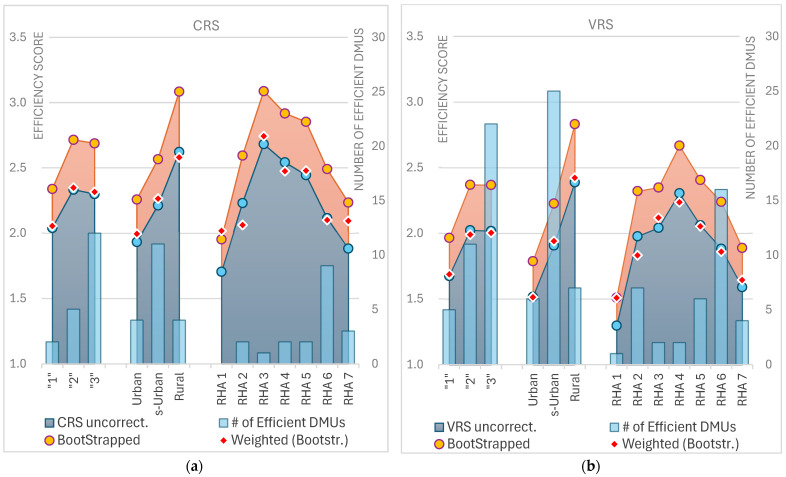
Comparison of the number of efficient Health Centers (HCs) and mean efficiency scores across regions. The graphs display the ordinary (uncorrected), bootstrapped (corrected), and weighted mean efficiency scores for (**a**) constant returns to scale (CRS) and (**b**) variable returns to scale (VRS). Regions are categorized according to Eurostat’s typology (‘1’, ‘2’, ‘3’), FAO’s classification (urban, peri-urban, rural), and Regional Health Authorities (RHA 1–7).

**Figure 6:**
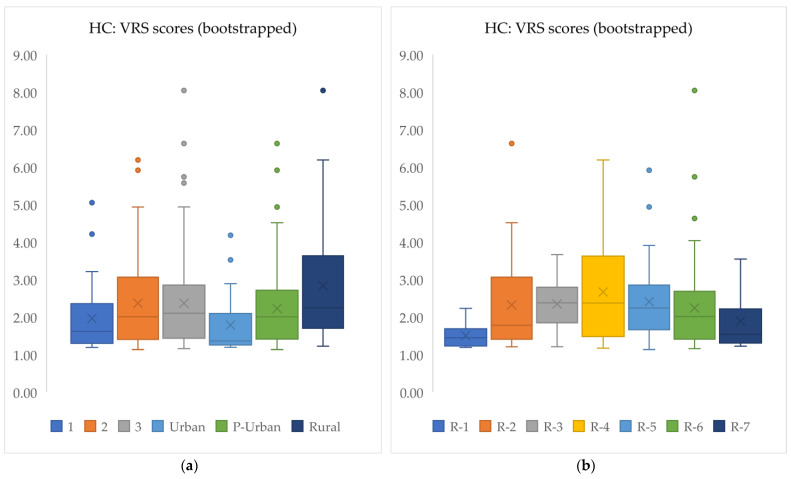
Boxplots of bootstrapped VRS efficiency scores for Health Centers (HCs) across regions. Panel (**a**) shows distributions categorized by Eurostat’s typology (‘1’, ‘2’, ‘3’) and FAO’s classification (urban, peri-urban, rural), while panel (**b**) presents distributions by Regional Health Authorities (RHA 1–7).

The overall uncorrected mean efficiency score across all regions was 1.97, indicating that, on average, units could nearly double their outputs while maintaining the same input levels. After applying bootstrapping, the bias-corrected mean efficiency score rose to 2.31, as expected, since standard DEA scores tend to be overly optimistic. A maximum score of 8.04 revealed significant inefficiency in some units, suggesting substantial potential for output increases. The weighted mean efficiency score of 1.92 reflected slightly better performance when output adjustments were considered. In Figure 5b, the ordinary, bootstrapped, and weighted mean VRS scores are depicted across all subdivisions, alongside the CRS scores shown in Figure 5a, while the boxplots in Figure 6 provide a visual representation of the distribution of bootstrapped VRS scores, illustrating the median, quartiles, and potential outliers.

Examining the regions, Region ‘1’ had the lowest weighted mean efficiency score, with 1.69, implying that its units could enhance their outputs by 69%. The region’s lower maximum value (5.05) and smaller standard deviation suggested more consistent performance. In contrast, while Region ‘3’ had a weighted mean efficiency score of 2.00, indicating higher inefficiency, 57.9% of the efficient units were still located in this region. However, the maximum value of 8.04 for Region ‘3’ highlighted notable inefficiencies among some units.

Regarding urbanization levels, urban areas exhibited the highest efficiency, with a weighted mean score of 1.52. The low standard deviation and maximum score of 4.18 reflected more consistent performance. Peri-urban areas showed higher inefficiency, with a weighted mean score of 1.94 and greater variation in performance, while rural areas exhibited the highest inefficiency, with a weighted mean score of 2.42. This indicates significant potential for output improvement across all non-urban areas.

At the level of Regional Health Authorities, RHA 1 showed the highest efficiency with a mean score of 1.30, meaning that outputs could be increased by 30%. However, this region had only one efficient unit, indicating that few units reached top performance. In contrast, RHA 6 had a mean score of 1.88, suggesting great potential for output improvement, even though it hosted 42.1% of all efficient units. RHA 4 had the highest bootstrapped mean score (2.67), indicating greater inefficiency and variability among its units.

Similarly, Table 6 shows the corresponding figures for ToMYs across the seven Regional Health Authorities (RHAs). Overall, the mean efficiency score was 1.74, indicating that, on average, units could increase their outputs by 74%. The bootstrapped mean rose to 1.98, and the maximum score of 10.55 highlighted significant inefficiencies in certain units. The weighted mean of 1.58 reflected slightly better performance when adjusted for outputs. Since the weighted mean efficiency scores accounted for output levels, the lower score suggests that, on average, larger or higher-output units tend to be more efficient than smaller ones.

**Figure 7:**
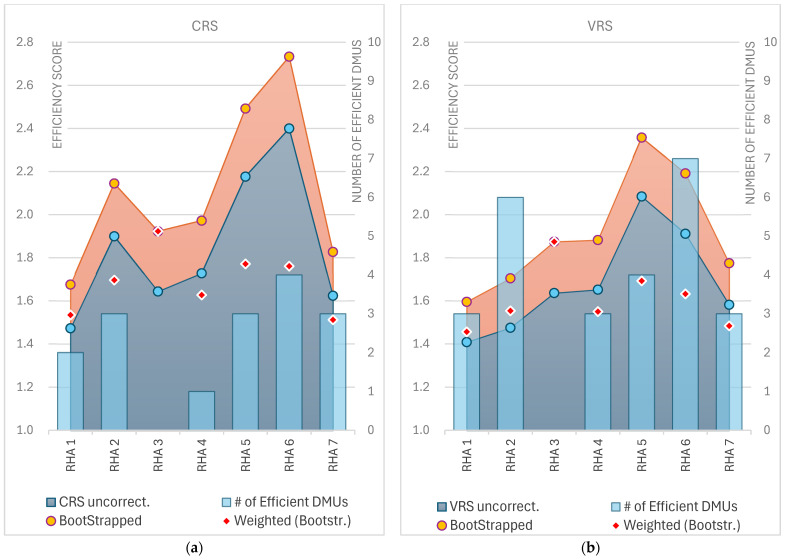
Comparison of the number of efficient ToMYs and mean efficiency scores across RHAs. The graphs display ordinary (uncorrected), bootstrapped (corrected), and weighted mean efficiency scores for (**a**) constant returns to scale (CRS) and (**b**) variable returns to scale (VRS) across Regional Health Authorities (RHA 1–7).

The standard deviation of 1.60 for RHA 5, one of the highest among RHAs, highlighted considerable variability in efficiency across its units. This suggested that while some units in RHA 5 performed near the average, others were significantly inefficient. Similarly, RHA 6 showed high variability, with a StDev of 1.83, further evidenced by its maximum score of 10.55, indicating significant disparities in efficiency within the region. In contrast, RHA 1 and RHA 7 exhibited more consistent performance, with lower standard deviations of 0.59 and 0.73, respectively, suggesting less variation in efficiency across their units. These findings suggest that regions with higher variability, such as RHA 5 and RHA 6, may require more targeted interventions to address inefficiencies, whereas RHA 1 and RHA 7 demonstrate more uniform, though still improvable, performance. In Figure 7, the mean values of ordinary (uncorrected) CRS/VRS scores and bias-corrected scores derived from Simar and Wilson’s bootstrap method are presented, both unweighted and weighted, for the seven Regional Health Authorities. The number of efficient facilities per RHA is shown as bars superimposed on the graph.

### 3.4. Super-Efficiency, Benchmarks, and Peer Counts

Table 7 presents the super-efficiency, output-oriented DEA scores for Health Centers, listing all of them that were super-efficient in at least one of the four models: (CRS), (VRS), (NIRS), and (NDRS). In an output-oriented framework, a super-efficiency score below 1 reflects the distance of a unit from the “reduced” efficiency frontier, which is formed by excluding that unit. In essence, this score indicates the extent of a unit’s superior performance relative to all other units that reside on the frontier. The DMUs in the table are ranked according to their super-efficiency scores in the VRS model, with additional columns showing peer counts and relative ranks (based on super-efficiency scores) across all four models.

First, The VRS super-efficiency scores ranged from 0.071 to 0.999, indicating a wide variability in the efficiency levels of the HCs. The highest-ranked unit in the VRS model was D-041, with a score of 0.071, indicating exceptional super-efficiency. Closely following was D-184, with a VRS score of 0.211, further demonstrating a high level of efficiency. Both units served as benchmarks for other inefficient units. However, they each acted as peers for only two other units, which may indicate that while they exhibit strong individual performance, their specific operational characteristics may not be as broadly applicable to improving the efficiency of other units.

HCs with high peer counts, such as D-173 (108 peers under CRS) and D-159 (67 peers), served as key benchmarks for other less efficient units. These HCs set the standard for performance, being frequently referenced by other units for comparison. In contrast, HC D-173, while ranking ninth in VRS with a score of 0.412, had a notable peer count of 61, indicating its significant influence as a benchmark. Similarly, D-159 (ranked 11th in VRS) and D-048 (ranked 10th) had significant peer counts, influencing 62 and 14 units, respectively, indicating their strategic importance within the system despite their mid-range VRS rankings.

Some DMUs, like D-048 and D-178, showed high super-efficiency scores despite lower peer counts, indicating that while less frequently used as benchmarks, their output performance was still exceptional. The variation in super-efficiency scores illustrates the differing capacities of DMUs to increase output, with units like D-238 (0.982) and D-176 (0.992) having limited room for further output increases compared to top-ranked units like D-173 and D-159. Overall, the analysis identified top performers and key benchmarks while highlighting the variation in super-efficiency across different models.

In the table, DMUs D-199 and D-212 have missing super-efficiency scores under the VRS model because the linear programming (LP) problem was not solvable, resulting in an infeasible solution. This issue typically arises when the DMU in question lies on the boundary of the efficiency frontier and removing it from the reference set causes the LP model to lack a feasible reference point for comparison. Essentially, this means that the DMU is located in an “area” where no other units can serve as a benchmark to evaluate its performance under the VRS assumption.

### 3.5. FDH Results

The Free Disposal Hull (FDH) scores presented in Table 8 provide a detailed analysis of output-oriented efficiency across different regions. Looking at all DMUs, the average FDH score was 1.34, indicating that, on average, DMUs operated 34% away from the efficient frontier, which implied a significant degree of inefficiency in terms of output generation. The bootstrapped mean score of 1.51 (with a standard deviation of 0.56) further highlighted the considerable variation in efficiency across DMUs, with some units significantly underperforming. Among the 234 DMUs analyzed, 115 were classified as efficient, yet only 35 of them served as benchmarks for the remaining 119 inefficient DMUs. The weighted mean efficiency of 1.39 reinforced the fact that although a large proportion of DMUs operated inefficiently, a few highly efficient units helped balance the overall efficiency.

Regionally, there were clear distinctions. Region ‘2’ demonstrated the highest mean FDH score (1.40), indicating relatively lower efficiency, with the bootstrapped mean score of 1.58 and the highest standard deviation of 0.67 reflecting significant variation in performance across DMUs within this region. Conversely, Region ‘1’ showed the lowest mean FDH score (1.25), making it the most efficient region on average. However, despite its higher efficiency, Region ‘1’ did not serve as a major source of benchmarks for other regions as it had a smaller number of efficient DMUs compared to Region ‘2’.

When comparing the different areas according to FAO classification, urban DMUs exhibited the highest efficiency, with a mean FDH score of 1.17, suggesting that urban centers, on average, need only a 17% increase in their current output levels to reach the efficiency frontier, making them the most efficient category. The bootstrapped scores for urban DMUs also showed the lowest variability (StDev = 0.31), implying greater consistency in efficiency across urban units. Suburban and rural DMUs had similar mean FDH scores of 1.34 and 1.41, respectively, but rural DMUs showed more variability in their performance, as indicated by the wide range of bootstrapped scores (from 1.13 to 3.45).

**Figure 8:**
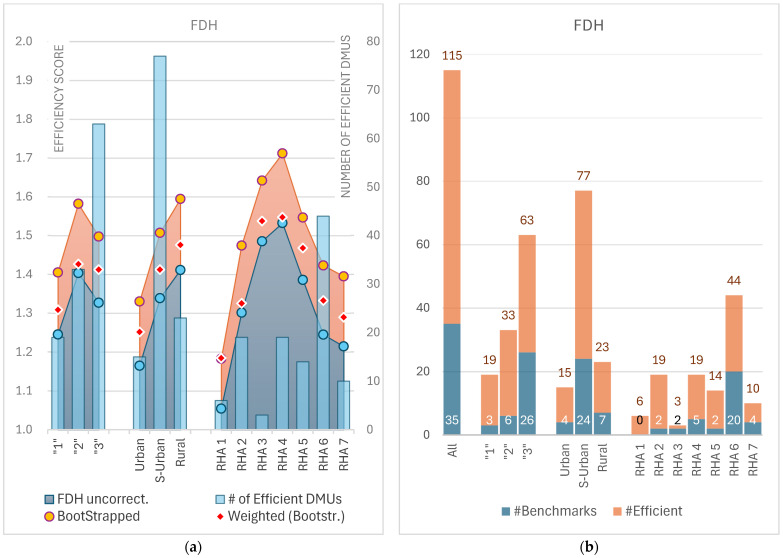
Distribution of Health Center (HC) FDH efficiency and benchmark units across regions, categorized by Eurostat’s typology (‘1’, ‘2’, ‘3’), FAO’s classification (urban, peri-urban, rural), and Regional Health Authorities (RHA 1–7). Panel (**a**) shows the number of efficient facilities alongside the ordinary (uncorrected), bootstrapped (corrected), and weighted mean FDH scores. Panel (**b**) presents the count and percentage of efficient HCs that served as benchmarks.

Among the Regional Health Authorities, RHA 4 and RHA 5 stood out for their relatively low efficiency, with mean FDH scores of 1.53 and 1.39, respectively. The high standard deviations in the bootstrapped scores for these regions suggested significant variation within them. On the other hand, RHA 1 was the most efficient, with a mean FDH score of 1.05 and very little variability (StDev = 0.10). Despite its high efficiency, RHA 1 did not provide any benchmark DMUs, indicating that it may not be heavily relied upon as a reference point for other inefficient DMUs in the dataset. Figure 8a presents the visualization of ordinary (uncorrected) FDH scores averaged across areas categorized by Eurostat’s typology (‘1’, ‘2’, ‘3’), FAO’s classification (urban, peri-urban, rural), and Regional Health Authorities (RHA 1–7), alongside the bootstrapped bias-corrected scores in both unweighted and weighted forms. Additionally, Figure 8b displays the total number of efficient facilities, along with the subset that served as benchmarks.

On an individual basis, Table 9 provides an overview of the 35 Health Centers identified as benchmark units under FDH, alongside the frequency of their appearances as peers for inefficient units. These data points are categorized by Eurostat regions (‘1’,’2’,’3’), FAO urbanization levels (urban, p[eri]-urban, rural), and Regional Health Authorities (RHA 1–7). The shaded cells in each row indicate the region of origin for the corresponding benchmark unit, offering a visual representation of the subcategory source and its respective RHA. The table also summarizes the total number of peer appearances for all benchmark units and the number of benchmarks within each subcategory.

D-037 stood out as the most frequently cited benchmark unit, appearing 19 times as a peer for inefficient Health Centers. This unit primarily originated from Region ‘3’ and peri-urban areas, with significant representation across five RHAs, particularly RHAs 3 and 5. Other notable benchmark units included D-260 and D-172, which appeared 11 and 10 times, respectively, and were also from Region ‘3’. The fact that Region ‘3’ contributed the largest number of benchmarks, with 26 units, indicates that this region hosts many of the most efficient production units, making it a focal point for operational learning and model replication. Conversely, Regions ‘1’ and ‘2’ had a smaller number of benchmark units, with only 3 and 6 benchmarks, respectively.

Peri-urban areas were the largest contributors, with 24 benchmark units, highlighting that these areas have found an optimal balance between operational complexity and resource management. This balance made peri-urban areas more likely to serve as benchmarks for less efficient units. By contrast, urban areas contributed only four benchmarks, while rural areas provided seven, suggesting that strategies implemented in peri-urban areas may be more adaptable and scalable across different settings. RHA 6 led in benchmark contributions with 20 units, signaling that its operational practices could serve as effective models for other RHAs. On the other hand, RHA 1, with no benchmarks, and RHA 7, with only four, showed room for significant improvement.

Similarly, Table 10 presents output-oriented FDH efficiency scores for ToMYs across the seven RHAs. The mean FDH score of 1.36 indicated a moderate level of inefficiency, suggesting that ToMYs have the potential to increase output by 36% with their existing resources. The bootstrapped mean score of 1.55, with a standard deviation of 0.81, highlighted significant variability in efficiency across DMUs. Bootstrapped scores ranged from 1.08 to 6.58, revealing both highly efficient and substantially inefficient units. Despite this, 52 DMUs were classified as efficient, though only 15 of these served as benchmarks for the remaining 42 units. The weighted mean efficiency score of 1.34 further supported the conclusion that while some DMUs perform well, there is considerable room for overall improvement.

RHA 2 stood out as the most efficient, with a mean FDH score of 1.16, indicating that ToMYs in this region operate near the efficiency frontier. Its bootstrapped mean of 1.33 and low standard deviation (0.41) further suggested consistent performance. In contrast, RHA 3 was the least efficient, with a mean FDH score of 1.64 and no efficient DMUs, indicating significant inefficiencies and a lack of benchmarks for other regions.

RHA 5 and RHA 6 also exhibited high inefficiency, with mean FDH scores of 1.61 and 1.45, respectively. RHA 6, in particular, showed a wide range of bootstrapped scores (from 1.10 to 6.58), highlighting significant disparities in performance among its units. Despite having the most efficient DMUs (18 units), only 4 of these served as benchmarks, suggesting that these high-performing units are underutilized for broader improvement efforts. In contrast, RHA 1 and RHA 2 demonstrated more consistent performance, with mean FDH scores of 1.25 and 1.16, respectively, and a higher proportion of efficient DMUs. Notably, in RHA 5, five benchmark units served as peers for 20 inefficient DMUs across all RHAs.

The 15 benchmark units are listed in Table 11 along with their frequency of appearance as peers for inefficient units across the seven RHAs. Again, the shaded cells indicate the region of their origin, and the total numbers of peer appearances and benchmarks per RHA are summarized at the bottom.

TM-047, originating from RHA 5, was the most frequently cited benchmark, appearing 10 times as a peer across all regions except 1 and 7. TM-021 (from RHA 2) followed with six appearances, spanning RHA 1, 2, 5 and 6. These benchmarks demonstrated high versatility and performance across regions.

RHA 5 emerged as a top performer, with five benchmark units and 20 peer appearances, indicating strong operational efficiency. RHA 6 also stood out, with four benchmark units that appeared six times as peers, though only one of these served as a peer within its own RHA, despite a total of 12 peer appearances.

### 3.6. Kruskal–Wallis Test

Table 12 presents the results of Kruskal–Wallis and median tests applied to all models—CRS, VRS, IRS, DRS, and FDH—both in their conventional form and bootstrapped versions. These tests assessed whether there were statistically significant differences across regions (‘1’, ‘2’, ‘3’), urbanization levels (urban, peri-urban, rural), and Regional Health Authorities (RHA 1–7). The Kruskal–Wallis test showed significant differences in the DRS model for regions, with a *p*-value of 0.05, indicating regional variations in efficiency with the decreasing returns to scale model. The bootstrapped DRS version showed near significance (*p* = 0.06), suggesting potential regional differences. For urbanization levels, both VRS and DRS models revealed significant differences, with *p*-values < 0.001, indicating strong efficiency variation between urban, peri-urban, and rural units, confirmed by the bootstrapped results. Similarly, the analysis of RHAs showed significant differences in the FDH (*p* = 0.01) and VRS (*p* = 0.02) models, reflecting efficiency variations across RHAs. Bootstrapped results for FDH, VRS, and DRS further reinforced these findings, with consistently significant *p*-values.

In the median test, the VRS model showed significant regional differences (*p* = 0.05), while the DRS model approached significance (*p* = 0.06), indicating potential regional efficiency variation. For urbanization levels, DRS showed significant differences (*p* = 0.01), suggesting that efficiency varied notably between urban, peri-urban, and rural units, which was confirmed by the bootstrapped results. Regarding RHAs, FDH (*p* = 0.01), VRS (*p* = 0.05), and DRS (*p* = 0.04) all showed significant differences, with bootstrapped results supporting these findings.

### 3.7. Scale Efficiency

The summary statistics of scale efficiency scores for Health Centers (HCs) and Local Health Centers (ToMYs) in Table 13 and Table 14 offered insights into how efficiently—on average—these units operated relative to their optimal size (MPSS). A score of 1 indicated full scale efficiency, while values greater than 1 suggested inefficiency, which could be caused by operating either above or below the optimal scale.

The mean scale efficiency score across all HCs was 1.17, showing that, on average, Health Centers operate at 17% inefficiency relative to their optimal scale. This inefficiency was further highlighted by the weighted mean of 1.19, suggesting that larger or higher output centers tend to be slightly less efficient overall. The median score of 1.05 indicated that most centers were relatively close to the optimal scale, but the maximum score of 2.71 revealed the presence of highly inefficient outliers, particularly in ‘3’/Rural/RHA 6 regions. Urban Health Centers exhibited the highest mean score of 1.30, reflecting the challenges of scale in larger, more complex centers. Conversely, peri-urban (mean 1.16) and rural centers (mean 1.12) demonstrated somewhat better efficiency, though rural centers still showed some inefficiencies, likely related to resource constraints in smaller, isolated populations.

Variability in scale efficiency was also notable. The standard deviation for all HCs was 0.27, with urban centers showing the highest variability (StDev = 0.33), highlighting substantial variations in the efficiency with which these larger centers operate at scale. Among the Regional Health Authorities, RHA 7 showed the highest variability (StDev = 0.35), while RHA 4 exhibited the least (StDev = 0.13), suggesting that some regions have more consistent performance while others vary greatly in efficiency.

The findings for Local Health Centers (ToMYs) presented a similar trend. The overall mean scale efficiency score for ToMYs was 1.20, whereas the weighted mean was lower, at 1.06. This discrepancy suggested that smaller or lower-output units may have been less efficient on average, pulling up the mean score, while larger or higher-output units tended to perform closer to the optimal scale, leading to a lower weighted mean score. The weighted mean accounted for the size and output of the units, emphasizing the efficiency of more productive ToMYs, and implied that although there were significant inefficiencies in the system overall, larger or busier ToMYs were more efficient.

Furthermore, the data in the table show that there was considerable regional variation. For RHA 2, the gap between the mean (1.39) and weighted mean (1.10) was particularly pronounced, indicating that smaller or less productive ToMYs in this region were significantly less efficient, skewing the mean upward. In contrast, the larger units in this region operated closer to their optimal scale. Similar trends could be observed for RHA 6, where the mean was 1.33, but the weighted mean was lower at 1.08, further reinforcing that larger ToMYs in this region were more scale-efficient compared to smaller ones. On the other hand, RHAs 1, 4, 5, and 7 showed minimal differences between their mean and weighted mean scale efficiency scores, indicating more uniform efficiency distribution across ToMYs in these regions. Both smaller and larger units appeared to perform similarly in terms of scale efficiency. Additionally, the fact that both the mean and weighted mean scores in these RHAs were close to 1 suggested that the units in these regions operated near their optimal scale.

### 3.8. Outlier Influence: Bias

Table 15 compares the efficiency characteristics of two samples: the 241 HCs sample (original dataset with 241 DMUs) and the 234 HCs sample (reduced dataset after removing seven outlier DMUs). This comparison illustrates the effects of outlier removal on efficiency scores and peer selection within a DEA VRS output-oriented model (where lower scores—being closer to 1—represent higher efficiency). The key findings are as follows.

In the 241 HCs sample, the mean efficiency score of all DMUs was 2.33, while, in the 234 HCs sample, it decreased significantly to 1.97, indicating a substantial improvement in overall performance. This finding suggests that the presence of outliers in the original sample inflated the efficiency scores across DMUs. Additionally, the total number of efficient DMUs increased from 31 in the 241 HCs sample to 38 in the 234 HCs sample. This change indicates that certain DMUs achieve efficiency only after the influence of outlier peers is removed, further highlighting the effect of outliers in skewing efficiency measurements and potentially introducing errors where truly efficient units may be misclassified as inefficient.

In the 241 HCs sample, four out of the seven total outliers were identified as efficient, while the remaining three were classified as inefficient. Removing these outliers helped prevent efficiency assessments from being disproportionately influenced by extreme values, resulting in a clearer distinction between efficient and inefficient DMUs. At first glance, it may seem unusual that the data cloud method flagged three DMUs as outliers, despite their inefficiency. However, closer examination of their scores—1.02, 1.01, and 1.09 for facilities D-009, D-011, and D-069, respectively—showed that they were very close to the efficient threshold. Although these DMUs were not classified as efficient in the initial analysis, their proximity to the (extreme) efficiency boundary caused a noticeable shift in the “cloud” volume when they were removed, leading to their classification as outliers. This finding, we believe, demonstrates the robustness of the data cloud method’s underlying concept.

However, a subset of 27 DMUs remained efficient in both samples, suggesting that these units maintained their efficiency status regardless of the presence of outliers. This consistency provided robustness to the analysis, indicating a core set of efficient DMUs that acted as stable benchmarks across sample variations.

In the 241 HCs sample, 183 DMUs used at least one efficient outlier as a peer, and 90.1% of inefficient DMUs relied on efficient outliers for benchmarking. This high dependence on outliers provides strong evidence of their substantial influence on the efficiency scores of other DMUs. Efficient outliers accounted for 32.1% of all peer occurrences in the 241 HCs sample, underscoring the significant role these outliers played in shaping the efficiency landscape. In the reduced 234 HCs sample, this reliance on outliers as peers was eliminated, potentially resulting in a more balanced and unbiased peer structure.

Finally, turning the lights on the subset of 180 DMUs common to both samples (those that used at least one efficient outlier as a peer in the 241 HCs sample), the mean efficiency score improved from 2.65 to 2.14 following outlier removal. This significant reduction indicated that outliers contributed to higher efficiency scores within this group. The standard deviation also decreased from 1.23 in the 241 HCs sample to 0.96 in the 234 HCs sample, suggesting a reduction in score variability and a more stable efficiency distribution without the influence of outliers.

Regarding extreme values, the minimum efficiency score of the 180 common DMUs subset approached 100% in the 234 HCs sample (100.0%), indicating that some DMUs achieved nearly optimal efficiency. The maximum score, however, decreased from 8.07 to 5.68, highlighting that removing outliers limited extreme values and provided a more conservative and realistic view of the efficiency range.

**Figure 9:**
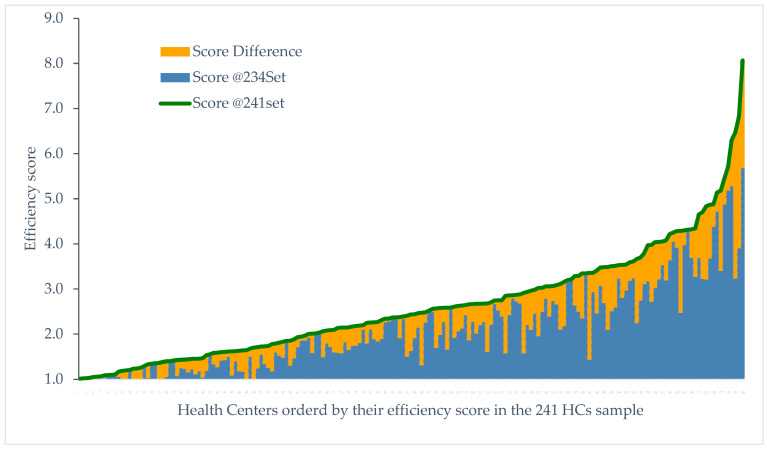
Efficiency scores for the subset of 180 common DMUs in ascending order, based on the 241 HCs sample (green line). The orange area represents score differences observed when calculated for the 234 HCs sample, illustrating the influence of removed outliers on efficiency score distribution.

In Figure 9, the efficiency scores of the subset of 180 common DMUs are presented in ascending order (green line), as calculated for the 241 HCs sample. The relative size of the orange area, representing the score differences of these DMUs when calculated within the 234 HCs sample, compared to the total area under the green line visually illustrates the extent of influence exerted by the removed outliers.

In summary, our findings demonstrate that outliers significantly impacted efficiency scores and peer relationships in the DEA model. By removing outliers, the 234 HCs sample provided a more reliable efficiency evaluation, reducing bias from questionably extreme performers. The more consistent range and reduced variability in the subset of 180 common DMUs underscored the benefits of this adjustment, suggesting that the 234 HCs sample offered a more accurate foundation for benchmarking and policy recommendations in healthcare efficiency.

## 4. Discussion

Strengthening primary healthcare (PHC) services is linked to improved equity in access, better clinical outcomes and population well-being, reduced healthcare and social costs, and increased productivity [3]. However, the growing healthcare needs of citizens—mainly due to increased life expectancy, population aging, technological innovations, and climate change—combined with limited available resources to meet these needs, make the efficient management of these resources imperative [32]. From a managerial perspective, understanding how scarce resources are utilized within the public healthcare system is essential for policymakers when making informed decisions [72].

In Greece, PHC development has been a central focus of reforms over the past decades, as it is considered essential for improving efficiency and access. To address these challenges, implementing modern methods to assess the efficiency of PHC units can provide invaluable insights to policymakers when developing health strategies. These methods facilitate the identification of inefficiencies and support corrective actions, contributing to ongoing performance monitoring and improvement. This study aimed to evaluate the efficiency of PHC units across Greece by assessing the efficiency levels of Health Centers (HCs) and Local Health Units (ToMYs) and identifying underlying factors contributing to observed inefficiencies. A variety of non-parametric methods were employed, primarily focusing on Data Envelopment Analysis (DEA), along with super-efficiency and Free Disposal Hull (FDH) techniques. Utilizing data from 2019, this study stands out as the only evaluation of Greek PHC units conducted after the financial crisis and before the COVID-19 pandemic.

The analysis of the data revealed significant inefficiency and differences in pure technical efficiency (VRS scores) between HCs and ToMYs. HCs, which operate with a much larger volume of activity, could nearly double their outputs while maintaining current input levels (mean weighted VRS bootstrapped score of 1.92), whereas ToMYs could increase their outputs by 58% (mean weighted VRS bootstrapped score of 1.58). This gap highlights the differing capacities of the two types of healthcare facilities to manage resources and serve their populations efficiently. The larger size and greater variability in outputs among HCs suggest more complex operational challenges, leading most likely to divergent operational profiles, which may be among the primary causes of their significant inefficiencies. In terms of scale efficiency, the scores showed better performance, with 1.17 for HCs and 1.20 for ToMYs, both very close to each other, indicating a slightly better proximity of HCs to their MPSS of operation.

Other findings from similar studies align with these observations. For instance, a study [34] of primary healthcare units in southern and western Greece revealed that technically efficient units were typically those covering larger populations and located near major cities. In contrast, less efficient units were in remote, sparsely populated areas that predominantly focused on acute medical services rather than preventive care. This mirrors the results of our study, where rural units generally exhibited higher inefficiencies, as these areas often face resource constraints and serve smaller populations.

In our analysis, HCs classified as ‘1’ and urban emerged as the most efficient (weighted mean VRS bootstrapped scores of 1.69 and 1.51, respectively), followed by the intermediate (‘2’) and peri-urban regions. Interestingly, the upward scaling of inefficiency was aligned along the transition from urban to rural areas in both categorization typologies (1 → 2 → 3, urban → peri-urban → rural). This finding, with respect to the FAO’s classification, was also found to be statistically significant according to the Kruskal–Wallis test.

The inefficiencies observed in rural and remote areas are consistent with the findings of one study [37] that reported that small, rural Health Centers, particularly those located on islands, exhibited lower efficiency, especially when lacking preventive care services. Similarly, another study [35] found that healthcare centers serving populations between 10,001 and 50,000 were the most efficient, whereas smaller centers serving fewer than 10,000 individuals demonstrated significantly lower efficiency. These findings suggest that an optimal population size, combined with proximity to urban centers, may enhance operational efficiency. Contrarily, other subsequent studies [4,36] highlighted that rural or remote and island-based health centers exhibited higher technical efficiency compared to urban and suburban centers, though the latter demonstrated superior scale efficiency. Our findings indicate that scale efficiency improves progressively from urban to rural areas.

Access to healthcare facilities is typically not a major issue for populations in large urban areas; however, residents of rural and remote regions face considerable barriers, often requiring them to travel to other areas or seek care from private practitioners. This may be attributed to the better staffing and equipment levels typically found in larger primary healthcare (PHC) units in urban areas. The challenges faced in rural settings are multifactorial, involving not only geographical and economic barriers but also differences in sociodemographic factors, perceptions, and healthcare needs, all of which influence access to and utilization of PHC services [4,6,73,74].

In our study, rural areas displayed the highest inefficiency, with a mean VRS score of 2.42, indicating the potential for a 142% increase in outputs. These findings underscore the persistent inefficiency challenges in rural settings, where geographic isolation, small population sizes, and limited resources present significant operational obstacles. This dichotomy was further emphasized in our results, where urban centers exhibited inefficiencies largely due to over-scaling, while semi-urban centers achieved a more balanced alignment between scale and resource management.

The findings across RHAs highlighted significant disparities in efficiency among them. RHA 1 emerged as the most efficient, with mean weighted VRS (pure technical) efficiency scores of 1.51 for its Health Centers (HCs) and 1.46 for its Local Health Units (ToMYs). However, the region had only a single HC classified as efficient, suggesting that top-level performance was achieved by only a small fraction of its facilities. On the other hand, RHA 3 and RHA 4 showed the highest inefficiency among HCs, with weighted mean scores of 2.12 and 2.24, respectively. RHA 6, despite contributing 42.1% of all efficient units, had a weighted mean score of 1.86, indicating significant room for output improvement. A previous study [34] reported a better efficiency level for RHA 6, suggesting that efficiency levels may have declined over time or that results may differ based on the methodologies used. Furthermore, examining the FDH results, RHA 6 accounted for 20 out of the 35 benchmark units (57%), offering strong evidence that its operational practices could serve as effective models not only for other RHAs but also for improving its own performance. On the other hand, RHA 1, with no benchmarks, and RHA 7, with only four, showed room for significant improvement. To address these disparities, it is recommended to replicate best practices from highly efficient areas in less efficient regions. Additionally, further investigation into outliers, particularly in rural areas, may reveal specific factors contributing to inefficiencies.

Regarding ToMYs, their VRS inefficiency levels across the seven RHA regions appeared to be more consistent, averaging around 1.60 and falling within a range of approximately 20 percentage points. Similarly, their FDH scores appeared to be consistent across all RHAs, remaining close to the overall weighted mean value (1.34). In terms of benchmarks, RHAs 5 and 6 accounted for the majority, hosting 5 and 4 out of the total 15, highlighting these regions as leaders in efficiency. Policymakers should focus on transferring the successful operational practices from these high-performing regions to underperforming areas. Doing so could substantially enhance the overall efficiency of the healthcare system.

In the context of super-efficiency results, several interesting points emerged from a policymaking perspective. First, the infeasibility of calculating super-efficiency VRS scores for DMUs D-199 and D-212 may suggest that these units are highly efficient and play a critical role in shaping the efficiency frontier. However, without a solvable super-efficiency score, it is difficult to directly compare their performance with other top-performing units. These units are considered hyper-efficient [75] and require a thorough examination to understand their unique operational performance. Policymakers might interpret this as an indication that D-199 and D-212 represent unique or specialized units within the dataset, potentially operating at an optimal level that cannot be surpassed by other DMUs under VRS. This finding should be considered alongside the fact that these two units had a notable presence as peers in the VRS model. As such, these units could serve as models of efficiency within the system, but their specific operational circumstances should be further analyzed to ensure sustained high performance. Second, certain DMUs such as D-041, and D-184 were ranked as super-efficient, yet they did not appear as peers for any inefficient units. This suggests that while these units operate exceptionally well, their specific input–output combinations may not be directly comparable to those of other DMUs, making them unsuitable as benchmarks. From a managerial perspective, this implies that these DMUs may be achieving efficiency through unique circumstances or specialized processes that are difficult to replicate. Although these DMUs excel in performance, their lack of peer appearances suggests limited potential for other units to model their practices, warranting further investigation into the factors driving their idiosyncratic efficiency.

The efficiency deficit identified in this study can be attributed to resource shortages, such as the understaffing of medical and nursing personnel, particularly general practitioners, due to inadequate financial incentives, educational programs, and career progression opportunities [4,9,76,77,78]. Additionally, there is an unequal distribution of healthcare professionals between urban and rural areas [8] and a lack of facilities and diagnostic equipment in primary healthcare (PHC) centers [5,6], which limits service delivery, particularly in rural areas [79,80].

These challenges lead to gaps in preventive and health promotion services [8], barriers to access, increased waiting times, citizen dissatisfaction [15,80], and unmet healthcare needs [74,81]. As a result, many individuals turn to private doctors [9,82,83], increasing out-of-pocket health costs, among the highest in the EU [15,84,85]. Greece’s hospital-centered system further contributes to inefficiency, with PHC services often provided through hospital emergency departments without mandatory referrals from PHC structures [5,15,86]. Though the EOPYY, established in 2011, aimed to reduce hospital overload by directing PHC cases to public units, patients frequently bypass PHC facilities and opt for hospitals, viewing the referral process as bureaucratic [74]. Mandatory referrals (gatekeeping system) from general practitioners, proven to increase efficiency and lower costs in other countries [87,88], are still not implemented in Greece, where general practitioners make up only 7% of the total physicians [84]. However, the COVID-19 pandemic saw more citizens utilizing PHC units to avoid going to hospital emergency departments [89].

Despite reforms, integration remains low, particularly in prevention and public health services [8], with Greece spending just 1.4% of its health budget on these areas, compared to the EU27 average of 2.9% [84]. A study in Crete and Epirus [81] revealed gaps in health promotion activities and the limited implementation of e-health, EHR, and telemedicine services. However, later, during the COVID-19 pandemic, the push for digitalization helped PHC units meet citizen needs, particularly through remote services [89,90,91].

In the present analysis, there are some limitations that should be considered. First, due to data inconsistency and/or unavailability, the results are based on only a portion of the total PHC facilities (76.2% of Health Centers and 80.3% of ToMYs), leaving out a significant portion of the total volume of inputs and outputs. Although this study included the majority of PHC facilities, providing a reliable overall picture, there may still be differences at an individual level and in conclusions drawn from aggregated indicators when referring to smaller groups/subdivisions. These variations could affect the interpretation of results for specific regions or units. Second, while the quantitative variations in technical efficiency were thoroughly addressed, equally important variations related to the quality of services offered were not captured due to a lack of necessary data. Ideally, the volume of outputs should be accompanied by a measure of service quality, as high efficiency scores do not necessarily imply high-quality outcomes. However, incorporating quality measures into DEA models presents certain challenges and limitations. This remains a critical area for future research, contingent upon the availability of relevant data.

## 5. Conclusions

The findings of this study revealed that there is room for improving the efficiency of primary healthcare units by expanding or strengthening the services offered to better meet healthcare needs.

Primary healthcare (PHC) serves as the gateway to the healthcare system, playing a crucial role in the effective treatment of patients and the efficient management of resources. These objectives are of central importance, especially in a context where healthcare needs are continuously rising, while available resources remain limited.

By broadening the range of outputs—through the development of new services and the enhancement of existing ones, in collaboration with the other services of the National Health System—PHC could increase its efficiency and make a significant contribution to addressing healthcare needs, alleviating the burden on hospitals and reducing wasteful use of healthcare resources.

## Figures and Tables

**Table 1:** Production possibility set (*PPS*) assumptions and constraints for different DEA and FDH models. Summary of assumptions and structural constraints for each *PPS*.

Scaling	Free Disposability	Convexity	Returns to Scale
$\Lambda \left(CRS\right)=\left\{\lambda_{r}\in \;R_{+}\;|\;\sum_{r=1}^{n}\lambda_{r}>0\right\}$	•	•	• (CRS)
$\Lambda \left(NIRS\right)=\left\{\lambda_{r}\in \;R_{+}\;|\;\sum_{r=1}^{n}\lambda_{r}\leq 1\right\}$	•	•	• (NIRS)
$\Lambda \left(NDRS\right)=\left\{\lambda_{r}\in \;R_{+}\;|\;\sum_{r=1}^{n}\lambda_{r}\geq 1\;\right\}$	•	•	• (NDRS)
$\Lambda \left(VRS\right)=\left\{\lambda_{r}\in \;R_{+}\;|\;\sum_{r=1}^{n}\lambda_{r}=1\right\}$	•	•	-
$\Lambda \left(FDH\right)=\left\{\lambda_{r}\in \;N_{+}\;|\;\sum_{r=1}^{n}\lambda_{r}=1\right\}$	•	-	-

**Table 2:** Cross-reference of PHC DEA efficiency analysis studies utilizing specified input–output variables, including data sources and relevant literature references.

Inputs			Outputs			Ref.
i1	i2	i3	o1	o2	o3	
•	•	•				[59]
•	•		•	•		[61]
•	•				•	[60]
•	•	•	•	•		[4]
•	•					[62]
•	•	•	•	•		[36]
•	•		•	•	•	[34]
•	•	•		•	•	[33] *
•	•	•				[35]
Data Sources						
MoH	MoH	MoH	MoH	MoH	MoH	

Note: (i1) No. of medical staff, (i2) no. of nursing and paramedical staff, (i3) no. of administrative and other staff, (o1) no. of scheduled patient visits, (o2) no. of emergency patient visits, (o3) no. of patient visits for obtaining pharmaceutical prescriptions. (*) Systematic review article, MoH = Ministry of Health.

**Table 3:** Descriptive statistics for input and output variables of Health Centers (HCs) and ToMYs.

Entity	Statistic	Inputs			Outputs		
		i1	i2	i3	o1	o2	o3
HCs (N = 234)							
	Mean	11.4	15.7	8.2	15,992.4	9255.6	10,409.4
	St Dev	8.7	10.0	4.9	13,975.1	8845.2	7185.5
	Median	8.0	13.5	7.5	11,917.0	6333.0	8529.5
	Min	1	1	1	341	75	364
	Max	47	53	31	75,381	54,156	36,929
	Range	46	52	30	75,040	54,081	36,565
	Sum	2665	3675	1908	3,742,211	2,165,821	2,435,798
ToMYs (N = 94)							
	Mean	2.6	3.1	2.5	4944.7	1025.9	2860.5
	St Dev	1.2	0.9	0.7	3123.5	845.2	2019.9
	Median	3.0	3.0	3.0	4491.0	786.0	2419.0
	Min	1	1	1	465	12	109
	Max	5	5	5	14,161	3812	8737
	Range	4	4	4	13,696	3800	8628
	Sum	242	293	234	464,801	96,438	268,890

Note: (i1) No. of medical staff, (i2) no. of nursing and paramedical staff, (i3) no. of administrative and other staff, (o1) no. of scheduled patient visits, (o2) no. of emergency patient visits, (o3) no. of patient visits for obtaining pharmaceutical prescriptions.

**Table 4:** Results of the global returns-to-scale (RTS) determination test for Health Centers (HCs) using a homogenous bootstrap approach (B = 2000 replications).

Test	Null vs.	Statistic	Significance Level	
	Alternative	(ts_1,_ ts_2_)	α = 0.01	α = 0.05
test-1	H_0_: CRS	1.1526	*p* < 0.001	*p* < 0.001
	H_1_: VRS			
test-2	H’_0_: NRS	1.0256	*p* = 0.005	*p* = 0.0025
	H’_1_: VRS			

**Table 5:** Summary statistics of Health Centers’ (HCs) VRS bootstrapped efficiency scores, conventional and bootstrapped weighted means, and number and percentage of efficient DMUs. Data are categorized by Eurostat’s typology (‘1’, ‘2’, ‘3’), FAO classification (urban, peri-urban, rural), and Regional Health Authorities (RHA 1–7).

Region	Mean	Bootstrapped Scores (B = 2000 repls.)					Efficient DMUs		Weighted Mean Eff.
		Mean	StDev	Median	Min	Max	No.	% Total	
All	1.97	2.31	1.13	2.02	1.13	8.04	38	100.00%	1.92
‘1’	1.67	1.97	0.92	1.62	1.19	5.05	5	13.20%	1.69
‘2’	2.02	2.37	1.21	2.01	1.13	6.19	11	28.90%	1.99
‘3’	2.02	2.37	1.13	2.10	1.16	8.04	22	57.90%	2.00
Urban	1.52	1.79	0.82	1.36	1.19	4.18	6	15.80%	1.51
P-Urban	1.91	2.23	0.98	2.01	1.13	6.63	25	65.80%	1.94
Rural	2.39	2.83	1.48	2.25	1.22	8.04	7	18.40%	2.42
RHA 1	1.30	1.51	0.32	1.45	1.19	2.23	1	2.60%	1.51
RHA 2	1.98	2.32	1.24	1.78	1.20	6.63	7	18.40%	1.83
RHA 3	2.04	2.35	0.72	2.38	1.21	3.67	2	5.30%	2.12
RHA 4	2.31	2.67	1.30	2.37	1.17	6.19	2	5.30%	2.24
RHA 5	2.06	2.41	1.07	2.24	1.13	5.92	6	15.80%	2.05
RHA 6	1.88	2.24	1.15	2.01	1.16	8.04	16	42.10%	1.86
RHA 7	1.59	1.89	0.77	1.54	1.22	3.55	4	10.50%	1.64

**Table 6:** Summary statistics of ToMYs’ VRS bootstrapped efficiency scores, conventional and bootstrapped weighted means, and number and percentage of efficient DMUs. Data are categorized by Regional Health Authorities (RHA 1–7).

RHA	Mean	Bootstrapped Scores (B = 2000 repls.)					Efficient DMUs		Weighted Mean Eff.
		Mean	StDev	Median	Min	Max	No.	% Total	
All	1.74	1.98	1.33	1.48	1.11	10.55	26	100.00%	1.58
RHA 1	1.41	1.60	0.59	1.32	1.13	3.03	3	11.50%	1.46
RHA 2	1.48	1.70	0.65	1.48	1.11	3.53	6	23.10%	1.55
RHA 3	1.64	1.87		1.87	1.87	1.87	0	0.00%	1.87
RHA 4	1.65	1.88	0.95	1.52	1.13	3.94	3	11.50%	1.55
RHA 5	2.08	2.36	1.60	1.59	1.14	6.54	4	15.40%	1.69
RHA 6	1.91	2.19	1.83	1.51	1.15	10.55	7	26.90%	1.63
RHA 7	1.58	1.77	0.73	1.38	1.16	3.03	3	11.50%	1.48

**Table 7:** Super-efficiency scores of efficient HCs, relative ranks, and peer counts as benchmarks under four different returns-to-scale (RTS) assumptions. The table is ordered in ascending order based on VRS ranks. Minimum and maximum values are denoted in bold.

DMU	Super-Eff CRS		Peer Count	Super-Eff VRS		Peer Count	Supper-Eff NDRS		Peer Count	Super-Eff NIRS		Peer Count
	Score	Rank		Score	Rank		Score	Rank		Score	Rank	
D-199	**0.956**	19	0	-	1	34	-	1	34	0.956	26	0
D-212				-	1	11	-	1	11			
D-041				**0.071**	3	2	**0.071**	3	2			
D-184				0.211	4	2	0.211	4	2			
D-168	0.838	10	5	0.310	5	3	0.310	5	5	0.838	14	3
D-043				0.314	6	0	0.314	6	0			
D-243				0.391	7	2	0.391	7	2			
D-153				0.393	8	16	0.393	8	16			
D-173	**0.476**	1	108	0.412	9	61	0.412	9	103	**0.476**	1	66
D-048	0.745	7	33	0.428	10	14	0.428	10	35	0.745	8	12
D-159	0.598	3	67	0.550	11	62	0.598	13	61	0.550	2	68
D-172	0.611	4	94	0.555	12	66	0.555	11	94	0.611	5	66
D-037	0.614	5	66	0.590	13	69	0.614	14	61	0.590	3	74
D-252	0.596	2	70	0.594	14	70	0.594	12	67	0.596	4	73
D-178	0.798	9	9	0.623	15	3	0.623	15	8	0.798	13	4
D-256	0.695	6	97	0.682	16	70	0.695	17	94	0.682	6	73
D-211				0.693	17	0	0.693	16	0			
D-035				0.739	18	31				0.739	7	31
D-058				0.774	19	29				0.774	9	29
D-093	0.795	8	22	0.775	20	16	0.775	18	22	0.795	12	16
D-189	0.868	11	5	0.780	21	1	0.868	21	4	0.780	10	2
D-166	0.871	13	59	0.781	22	81	0.871	22	56	0.781	11	84
D-111	0.875	14	2	0.791	23	3	0.791	19	2	0.875	19	3
D-044				0.842	24	8				0.842	15	8
D-225	0.869	12	32	0.852	25	18	0.852	20	35	0.869	18	14
D-057	0.879	16	43	0.853	26	33	0.879	25	38	0.853	16	38
D-007				0.858	27	9				0.858	17	9
D-260	0.876	15	48	0.875	28	23	0.876	24	47	0.875	20	24
D-162	0.888	17	7	0.876	29	1	0.876	23	5	0.888	23	3
D-028				0.885	30	21				0.885	21	21
D-155	0.920	18	3	0.885	31	5	0.920	26	1	0.885	22	7
D-203				0.910	32	25				0.910	24	25
D-120				0.924	33	8				0.924	25	8
D-262				0.959	34	1	**0.959**	27	1			
D-133				0.965	35	3				0.965	27	3
D-238				0.982	36	3				0.982	28	3
D-176				0.992	37	1				0.992	29	1
D-152				**0.999**	38	2				**0.999**	30	2
# DMUs		19			38			27			30	

Note: Min and max scores are shown in bold.

**Table 8:** Summary statistics of Health Centers’ (HCs) FDH bootstrapped efficiency scores, conventional and bootstrapped weighted means, and number and percentage of efficient and benchmark DMUs. Data are categorized by Eurostat’s typology (‘1’, ‘2’, ‘3’), FAO classification (urban, peri-urban, rural), and Regional Health Authorities (RHA 1–7).

Region	Mean	Bootstrapped Scores (B = 2000 repls.)					Efficient DMUs		Benchmarks		Weighted Mean Eff.
		Mean	StDev	Median	Min	Max	No.	% Total	No.	Peer Count	
All	1.34	1.51	0.56	1.22	1.09	4.28	115	100.0%	35	119	1.39
‘1’	1.25	1.41	0.45	1.20	1.10	2.89	19	16.5%	3	21	1.31
‘2’	1.40	1.58	0.67	1.22	1.09	4.28	33	28.7%	6	12	1.43
‘3’	1.33	1.50	0.52	1.22	1.09	3.47	63	54.8%	26	86	1.41
Urban	1.17	1.33	0.31	1.20	1.10	2.26	15	13.0%	4	24	1.25
P-Urban	1.34	1.51	0.57	1.22	1.09	4.28	77	67.0%	24	68	1.41
Rural	1.41	1.59	0.60	1.22	1.13	3.45	23	20.0%	7	27	1.48
RHA 1	1.05	1.18	0.10	1.15	1.11	1.44	6	5.2%	0	0	1.18
RHA 2	1.30	1.47	0.55	1.21	1.10	3.44	19	16.5%	2	22	1.33
RHA 3	1.49	1.64	0.47	1.68	1.13	3.07	3	2.6%	2	2	1.54
RHA 4	1.53	1.71	0.69	1.33	1.09	3.45	19	16.5%	5	11	1.55
RHA 5	1.39	1.55	0.67	1.25	1.09	4.28	14	12.2%	2	4	1.47
RHA 6	1.24	1.42	0.43	1.22	1.09	3.31	44	38.3%	20	63	1.33
RHA 7	1.21	1.39	0.47	1.21	1.12	2.69	10	8.7%	4	17	1.29

**Table 9:** Frequency distribution of Free Disposal Hull (FDH) benchmark appearances as peers for Health Centers (HCs) across regions, categorized by Eurostat’s typology (‘1’, ‘2’, ‘3’), FAO’s classification (urban, peri-urban, rural), and Regional Health Authorities (RHA 1–7).

Count	Benchmark	Number of Benchmark Appearances as Peers													
		All	‘1’	‘2’	‘3’	Urban	P-Urban	Rural	Regional Health Authority						
									1	2	3	4	5	6	7
1	D-037	19	5	3	11	·	18	1	1	2	5	4	5		2
2	D-260	11		4	7		6	5		4		2		4	1
3	D-172	10		3	7		8	2		1	2	2	1	4	
4	D-203	9		3	6	1	8				1	4	1	3	
5	D-173	8		2	6		5	3					1	7	
6	D-166	6		3	3		6			1	1	1	2	1	
7	D-225	5		2	3	2	2	1				3	1	1	
8	D-178	4		1	3		1	3		1			1	2	
9	D-252	4	3	·	1		3	1	1			3			·
10	D-028	3		2	1		3			·			2		1
11	D-091	3			3		3			2	1	·			
12	D-092	3	1		2		3			1		·	1	1	
13	D-152	3		1	2	1	2				1	1	1		
14	D-104	2			2		1	1				·	1	1	
15	D-118	2		1	1	1	1				1	·		1	
16	D-208	2		2	·		1	1				1		1	
17	D-210	2			2	1	1						2	·	
18	D-222	2		1	1		2	·					1	1	
19	D-223	2			2		·	2						2	
20	D-232	2		·	2		1	1				1		·	1
21	D-236	2		1	1		·	2					1	1	
22	D-238	2	2		·	2			1			1		·	
23	D-057	1	·	1			1				·		1		
24	D-058	1	1				1		1		·				
25	D-088	1			1		·	1		1		·			
26	D-159	1		1	·	1							·	1	
27	D-162	1			1			1						1	
28	D-168	1			1			1					1	·	
29	D-176	1			1		1			1				·	
30	D-183	1			1		1				1			·	
31	D-211	1		·	1		·	1				1		·	
32	D-216	1		·	1		1						1	·	
33	D-233	1			1		·	1					1	·	
34	D-250	1			1		1	·						1	·
35	D-253	1	1	·			1	·				1			·
Total	Appearences	119	13	31	75	9	82	28	4	14	13	25	25	33	5
	Benchmarks	35	3	6	26	4	24	7	0	2	2	5	2	20	4

Note: The shaded cells in each row indicate the sub(category) of origin (i.e., column header) for the corresponding benchmark unit.

**Table 10:** Summary statistics of ToMYs’ FDH bootstrapped efficiency scores, conventional and bootstrapped weighted means, and number and percentage of efficient and benchmark DMUs. Data are categorized by Regional Health Authorities (RHA 1–7).

Region	Mean	Bootstrapped Scores (B = 2000 repls.)					Efficient DMUs		Benchmarks		Weighted Mean Eff.
		Mean	StDev	Median	Min	Max	No.	% Total	No.	Peer Count	
All	1.36	1.55	0.81	1.21	1.08	6.58	52	100.0%	15	42	1.34
RHA 1	1.25	1.42	0.39	1.22	1.14	2.25	6	11.5%	2	6	1.34
RHA 2	1.16	1.33	0.41	1.21	1.08	2.83	9	17.3%	2	7	1.27
RHA 3	1.64	1.82		1.82	1.82	1.82	0	0.0%	0	0	1.82
RHA 4	1.30	1.47	0.66	1.20	1.09	3.47	7	13.5%	1	1	1.31
RHA 5	1.61	1.83	1.06	1.22	1.16	4.12	8	15.4%	5	20	1.43
RHA 6	1.45	1.63	1.05	1.20	1.10	6.58	18	34.6%	4	6	1.34
RHA 7	1.25	1.41	0.36	1.24	1.15	2.08	4	7.7%	1	2	1.28

**Table 11:** Frequency distribution of Free Disposal Hull (FDH) benchmark appearances as peers for ToMYs across Regional Health Authorities (RHA 1–7).

Count	Benchmark	Number of Benchmark Appearances as Peers							
		ALL	Regional Health Authority (RHA/YPE)						
			1	2	3	4	5	6	7
1	TM-047	10		1	1	3	2	3	
2	TM-021	6	2	2			1	1	
3	TM-010	4	·				2		2
4	TM-051	4					2	2	
5	TM-054	4		1		1	·	2	
6	TM-073	3		2				1	
7	TM-006	2	1	1					
8	TM-091	2						1	1
9	TM-016	1		·				1	
10	TM-042	1				1			
11	TM-045	1					·	1	
12	TM-048	1	1				·		
13	TM-064	1	1					·	
14	TM-069	1						·	1
15	TM-084	1		1				·	
Total	Appearances	42	5	8	1	5	7	12	4
	Benchmarks	15	2	2	0	1	5	4	1

Note: The shaded cells in each row indicate the sub(category) of origin (i.e., column header) for the corresponding benchmark unit.

**Table 12:** Kruskal–Wallis and median test results for HCs across DEA and FDH (conventional and bootstrapped) models. Tests assessed efficiency score differences across regions, categorized by Eurostat’s typology (‘1’, ‘2’, ‘3’), FAO’s classification (urban, peri-urban, rural), and Regional Health Authorities (RHA 1–7).

(Sub)Categories		CRS	VRS	IRS	DRS	FDH	Bootstrapped				
Tested							CRS	VRS	IRS	DRS	FDH
Kruskal–Wallis test											
‘1’, ‘2’,’3’	*χ*^2^ (df = 2)	1.97	3.89	0.93	5.92	1.56	2.31	4.80	1.52	5.76	4.85
	*p*-value	0.37	0.14	0.63	0.05	0.46	0.32	0.09	0.47	0.06	0.09
Urban, P-Urban, Rural	*χ*^2^ (df = 2)	6.05	12.40	3.17	18.28	3.28	6.15	16.34	4.33	19.10	5.03
	*p*-value	0.05	0.00	0.21	0.00	0.19	0.05	0.00	0.12	0.00	0.08
RHA 1, 2, 3, 4, 5, 6, 7	*χ*^2^ (df = 6)	10.60	15.20	12.52	14.89	16.37	9.05	14.59	9.52	14.40	16.95
	*p*-value	0.10	0.02	0.05	0.02	0.01	0.17	0.02	0.15	0.03	0.01
Median test											
‘1’, ‘2’, ‘3’	*χ*^2^ (df = 2)	1.65	5.98	1.65	5.54	1.65	1.39	5.98	1.39	5.54	4.23
	*p*-value	0.44	0.05	0.44	0.06	0.44	0.50	0.05	0.50	0.06	0.12
Urban, P-Urban, Rural	*χ*^2^ (df = 2)	0.71	5.76	0.35	10.47	2.00	1.28	9.32	1.28	10.47	3.63
	*p*-value	0.70	0.06	0.84	0.01	0.37	0.53	0.01	0.53	0.01	0.16
RHA 1, 2, 3, 4, 5, 6, 7	*χ*^2^ (df = 6)	8.12	10.87	8.05	11.45	15.92	8.12	12.55	8.11	13.05	13.79
	*p*-value	0.23	0.09	0.23	0.08	0.01	0.23	0.05	0.23	0.04	0.03

**Table 13:** Summary tsatistics of scale efficiency scores for Health Centers (HCs) across regions categorized by Eurostat’s typology, FAO’s classification, and Regional Health Authorities (RHA 1–7).

Statistic	All	‘1’	‘2’	‘3’	Urban	P-Urban	Rural	Regional Health Authority (RHA/YPE)						
								1	2	3	4	5	6	7
Mean	1.17	1.23	1.18	1.15	1.30	1.16	1.12	1.34	1.17	1.29	1.09	1.18	1.15	1.21
StDev	0.27	0.29	0.29	0.25	0.33	0.25	0.28	0.31	0.29	0.32	0.13	0.25	0.28	0.35
Median	1.05	1.09	1.06	1.05	1.24	1.05	1.04	1.24	1.05	1.15	1.05	1.09	1.04	1.04
Min	1.00	1.00	1.00	1.00	1.00	1.00	1.00	1.03	1.00	1.00	1.00	1.00	1.00	1.00
Max	2.71	2.12	2.44	2.71	1.98	2.44	2.71	1.80	2.44	2.12	1.76	2.12	2.71	2.12
Weighted mean	1.19	1.24	1.21	1.15	1.35	1.17	1.08	1.36	1.15	1.25	1.11	1.19	1.16	1.30

Note: Classifications ‘1’, ’2’, and ’3’ according to Eurostat (‘1’ = urban areas, ‘2’ = intermediate areas, ‘3’ = rural areas); classifications ‘urban’, ‘p[eri]-urban’, and ‘rural’ according to FAO.

**Table 14:** Summary statistics of scale efficiency scores for ToMYs across Regional Health Authorities (RHA 1–7).

Statistic	All	Regional Health Authority (RHA/YPE)						
		1	2	3	4	5	6	7
Mean	1.20	1.04	1.39	1.00	1.06	1.04	1.33	1.02
StDev	0.66	0.07	1.07		0.08	0.04	0.81	0.03
Median	1.02	1.00	1.05	1.00	1.00	1.03	1.03	1.01
Min	1.00	1.00	1.00	1.00	1.00	1.00	1.00	1.00
Max	5.43	1.22	5.43	1.00	1.25	1.16	4.02	1.07
Weighted mean	1.06	1.04	1.10	1.00	1.05	1.03	1.08	1.01

**Table 15:** Comparison of efficiency metrics and peer relationships in two samples (241 HCs and 234 HCs) before and after the removal of outlier decision-making units (DMUs).

Entity	241 HCs Sample (Original)	234 HCs Sample (Outliers Removed)
Number of decision-making units (DMUs) in sample	241	234
Number of outliers	7	-
Number of efficient DMUs	31	38
Number of efficient outliers	4	-
Number of inefficient outliers	3	-
Number of efficient DMUs common to both samples	27	27
Number of DMUs with at least one efficient outlier as a peer	183	-
Percentage of inefficient DMUs using at least one efficient outlier as a peer	90.1%	-
Total occurrences of efficient outliers as peers	261	-
Total occurrences of all efficient DMUs as peers	812	-
Percentage of peer occurrences for efficient outliers (of total)	32.1%	-
Mean efficiency score of all DMUs in sample	2.33	1.97
Number of inefficient DMUs common to both samples (using at least one efficient outlier as a peer in the 241 HCs sample)	180	180
Descriptive statistics of the subset (180 common DMUs)	241 HCs Sample	234 HCs Sample
Mean efficiency score	2.65	2.14
Standard deviation	1.23	0.96
Minimum efficiency score	1.02	1.00
Maximum efficiency score	8.07	5.68

## Data Availability

Restrictions apply to the availability of these data. Data were obtained from the Ministry of Health (Greece) and are available from the corresponding author with the permission of MoH.
